# Functional characterization of FAM120A as a novel effector in the progranulin/EphA2 oncogenic axis in bladder cancer

**DOI:** 10.1186/s13046-026-03808-1

**Published:** 2026-08-11

**Authors:** Vrunda Satasiya, Canio Martinelli, Gabriel Pascal, Giacomo Ducci, Elisa Ventura, Stephen J. Williams, Sharon Raffaella Burk, Manuela Nana Tchamou, Shendi Shani, Michele Klain, Elena Sacco, Marco Vanoni, Antonino Belfiore, Renato V. Iozzo, Antonio Giordano, Andrea Morrione

**Affiliations:** 1 Sbarro Institute for Cancer Research and Molecular Medicine, Center for Biotechnology, Department of Biology, College of Science and Technology, Temple University, Philadelphia, PA USA; 2 Department of Medical Biotechnologies, University of Siena, Siena, Italy; 3 Department of Human Pathology of Adult and developmental Age, Unit of Gynecology and Obstetrics, Gaetano Martino University Hospital, Messina, Italy; 4 Department of Pathology and Genomic Medicine, and the Translational Cellular Oncology Program, Sidney Kimmel Cancer Center, Thomas Jefferson University, Philadelphia, PA USA; 5 Mayo Clinic Alix School of Medicine, Phoenix, AZ USA; 6 Department of Biotechnology and Biosciences, University of Milano-Bicocca, Milan, Italy; 7 S.H.R.O. Italia Foundation ETS, Candiolo, Turin, Italy; 8 Department of Advanced Biomedical Sciences, University of Naples Federico ll, Naples, Italy; 9 Department of Clinical and Experimental Medicine, Endocrinology Unit, University of Catania, Garibaldi-Nesima Hospital, Catania, Italy

**Keywords:** FAM120A, EphA2, Progranulin, Bladder cancer, Migration, Invasion, Anchorage-independent growth

## Abstract

**Background:**

Bladder cancer (BC) is one of the most deadly diseases in the USA, with 84,530 new cases and 17,870 estimated deaths in 2026. The growth factor progranulin is involved in several human pathologies, including frontotemporal dementia (FTD), immune response, and cancer. We showed that in BC, progranulin and its signaling receptor, EphA2, drive tumor cell motility, invasion, and *in vivo* tumor formation, making it a critical pathway in tumor establishment. However, the molecular mechanisms of progranulin/EphA2 action are still poorly defined.

**Methods:**

Progranulin-dependent EphA2 interactome was explored by proteomic approaches. FAM120A or EphA2 protein levels in BC tissue microarrays (TMAs) and FAM120A protein expression in BC cell lines were analyzed by immunohistochemistry (IHC) and immunoblots, respectively. Progranulin-dependent activation of AKT and ERK1/2 were examined by western immunoblots. EphA2-FAM120A interaction was detected using Co-immunoprecipitation and proximity ligation assays (PLA). In addition, EphA2 and FAM120A colocalization and F-actin cytoskeleton rearrangements were assessed by immunofluorescence. The biological function of FAM120A was determined using lentiviral shRNA approaches, wound healing, motility and invasion, spheroid, soft agar, clonogenic assays, cell cytotoxicity, and *in vivo* xenograft models.

**Result:**

We conducted proteomic approaches and identified novel progranulin-dependent EphA2 interactors, including FAM120A, which is a scaffold protein with a putative role in oncogenic pathways. We confirmed that the EphA2 and FAM120A interaction was enhanced upon progranulin stimulation, and FAM120A was upregulated in BC tissues. We further demonstrated that FAM120A was critical for progranulin-evoked AKT and ERK1/2 activation, clonogenic capacity, wound healing, motility, invasion, and 3D spheroid formation of BC cell lines. In addition, FAM120A was essential for anchorage-independent growth and *in vivo* tumorigenesis in xenograft models. In addition, FAM120A depletion sensitized BC cells to cisplatin treatment. Mechanistically, we showed that FAM120A depletion inhibited progranulin-dependent F-actin cytoskeleton rearrangement through ERK1/2 and RhoA-dependent pathways. Furthermore, we demonstrated that progranulin-induced activation of RhoA was inhibited upon FAM120A depletion.

**Conclusion:**

The discovery of FAM120A as an oncogenic progranulin-dependent EphA2 interactor provides novel insights into the progranulin/EphA2 signaling axis and uncovers putative novel targets for BC therapy. Furthermore, FAM120A expression may work as a biomarker with diagnostic and possibly prognostic value in BC.

**Supplementary Information:**

The online version contains supplementary material available at https://doi.org/10.1186/s13046-026-03808-1.

## Background

Bladder cancer (BC) is among the most common cancer types in the United States. In 2026, the American Cancer Society has estimated about 84,530 new cases and approximately 17,870 deaths in the United States [1]. These statistics demonstrate the major impact of this malignancy on public health and the burden of care for affected individuals and survivors, as in fact BC is associated with substantial morbidity and mortality due to the burden of treatment and the cost of supportive care for this malignancy [2]. Despite its considerable epidemiological and economic impact, BC remains underrepresented in research prioritization and funding allocation compared to other high-mortality malignancies, including hepatocellular, gastric, and pancreatic cancers, or those characterized by complex clinical management and substantial healthcare expenditures. Moreover, despite intense investigation, most patients are diagnosed with highly aggressive, advanced-stage BC.

Progranulin is a pleiotropic growth factor which plays a critical role in several human pathologies, including frontotemporal dementia, immune response, and cancer [3–5]. The oncogenic progranulin precursor is a cysteine-rich protein encoded by the *GRN* gene in humans, and it is comprised of 7 (A-G) and a half (P) granulin motifs, which are stabilized by disulfide bonds [6], with a molecular weight of about 68.5 kDa. However, it is secreted as a highly glycosylated form of 88kDa. The progranulin precursor can be cleaved by proteases including elastases, matrix metalloproteases (MMPs), proteinase 3 [7], and ADAMTS-7 [8, 9], producing biologically active granulins which usually evoke opposing functions to the progranulin precursor protein [6]. Progranulin deficiency has been implicated in several neurodegenerative disorders, including frontotemporal dementia (FTD) and neuronal ceroid lipofuscinosis (NCL), and has also been linked to Alzheimer’s and Parkinson’s diseases [10, 11]. We previously demonstrated that progranulin promotes BC cell motility and invasion [12, 13] and regulates F-actin cytoskeletal remodeling through its interaction with the F-actin–binding protein, drebrin, which in turn modulates *in vivo* tumor growth.[14]. Moreover, depletion of progranulin markedly reduces tumor formation in both orthotopic and subcutaneous BC xenograft models and sensitizes urothelial cancer cells to cisplatin treatment [15]. Furthermore, we established that progranulin stimulates migration, invasion, proliferation, and anchorage-independent growth in prostate cancer [16] and also regulates migration, invasion, adhesion, and *in vivo* tumorigenesis in mesothelioma [17].

To better understand the molecular mechanisms underlying progranulin signaling, we previously identified EphA2 as a functional receptor for progranulin in BC [18]. Progranulin binding to EphA2 evokes the activation of AKT/ERK1/2 pathways and phosphorylation on S897 of EphA2, resulting in increased tumor cell motility and invasion capabilities [18, 19]. We recently used proteomic approaches [19] and characterized a novel progranulin-dependent EphA2 interactor, liprinα−1[19], which upon progranulin stimulation, modulates cell motility and invasion by regulating the dynamic assembly and disassembly of a plasma membrane–associated complex involved in migration. This complex contains paxillin along with liprinα−1 and/or liprinβ−1, vinculin, and ERC1/ELKS. Importantly, these four components were not previously associated with EphA2 and function collectively to enhance adhesion and invasion turnover, thereby promoting the formation of protrusions in migrating cells [19], making it an important regulator of the progranulin/EphA2 axis in BC. Although these studies established EphA2 as a critical mediator of progranulin signaling, the downstream protein networks that couple receptor activation to cytoskeletal remodeling, oncogenic signaling, and tumor progression are still poorly characterized. Identifying additional EphA2-associated effectors is therefore essential for better defining the molecular basis of progranulin-driven BC progression.

Among the candidate proteins identified through our proteomic analysis, FAM120A emerged as a particularly compelling molecule. FAM120A is a multifunctional scaffold and RNA-binding protein that orchestrates the assembly of signaling complexes involved in cell survival, cytoskeletal remodeling, RNA metabolism, oxidative stress responses, and receptor-mediated signal transduction [20, 21]. Initially identified as an activator of Src family kinases during oxidative stress, FAM120A facilitates PI3K/AKT/mTOR and FAK signaling by functioning as a molecular scaffold that recruits signaling components into receptor-associated complexes, thereby promoting cancer cell migration, invasion, survival, and metastatic dissemination [22]. More recently, FAM120A has also been implicated in mTORC1-dependent metabolic reprogramming and in the development of chemotherapy resistance through regulation of stress granule dynamics and RNA processing [20, 21]. Consistent with these diverse functions, elevated FAM120A expression has been reported in several human malignancies and is associated with aggressive tumor behavior, highlighting its role as a central signaling hub in cancer progression. These observations led us to hypothesize that FAM120A could function as a previously unrecognized downstream effector of the progranulin/EphA2 signaling axis in BC.

Here, using quantitative proteomics and functional analyses, we identify FAM120A as a novel progranulin-dependent EphA2-interacting protein in BC. We demonstrate that FAM120A is required for progranulin-mediated activation of oncogenic signaling, cytoskeletal remodeling, migration, invasion, and tumorigenesis. Furthermore, we show that FAM120A is overexpressed in human BC tissues, supporting its potential as a biomarker and therapeutic target in BC.

## Materials and methods

### Cell culture and reagent

Urothelial carcinoma-derived RT4, RT112, 5637, T24, HT1376, and UMUC-3 cells were obtained either by ATCC or the Cell Bank of the Fox Chase Cancer Center and maintained in RPMI (Corning, NY, USA) supplemented with 10% FBS (Phoenix Scientific) and 1% P/S. Immortalized SV-HUC-1 Bladder cells derived from uroepithelium were obtained from ATCC and maintained in Ham’s F-12K (Kaighn’s) Medium (Thermo Fischer Scientific) supplemented with 10% FBS (Phoenix Scientific) and 1% P/S.

Serum-free medium (SFM) is DMEM supplemented with 1% P/S, 0.1% bovine serum albumin, and 50 μg/ml of transferrin (Sigma-Aldrich, St Louis, MO, USA).

Human recombinant His-tagged progranulin was purified as previously described [23].

RhoA inhibitor, Rhosin (30μM) (Sigma), Rac1 inhibitor, EHT 1864 (50μM) (MedChemExpress), AKTi VIII (12.5μM) (MedChemExpress), and Trametinib (100nM) (CellChem) were used at the indicated concentrations.

### EphA2 pull-down assays and proteomic analysis

Proteomic analysis was performed as previously described [19]. Briefly, serum-starved T24 cells were treated with or without progranulin (150 nM) for 15 minutes, and lysates were immunoprecipitated with anti-EphA2 antibodies (Cell Signaling Technology), and complexes were pulled down using Agarose beads. Mass Spectrometry analysis was performed at the CCSG proteomics and metabolomics facility of the Wistar Institute. Fold change was calculated using the spectral count ch1 and PGRN P1 shown in additional data file 2.

### Co-Immunoprecipitation and immunoblotting

Serum-starved cells were stimulated with or without 100 nM progranulin, and lysates were collected using JS1X lysis buffer (HEPES 50 mM, NaCl 150 mM, Glycerol 1%, Triton X-100 1%, MgCl_2_ 1.5 mM, EGTA pH 8 5 uM) supplemented with Halt Protease and phosphatase inhibitors cocktail (Thermo Fisher Scientific). Lysates (5 mg) were precleared in protein-A Agarose beads (Santa Cruz Biotechnologies) for 1 hour at 4 °C on a rocking platform and then incubated with anti-EphA2 antibody (Cat. No, 6997, Cell Signaling Technologies) or anti-FAM120A antibody (Cat. No. A303-889A, Fortis Life Sciences) ON at 4°C. Immunocomplexes were pulled down by incubating with protein-A Agarose for 1 hour at 4 °C on a rocking platform.

Proteins were detected by immunoblots using the following antibodies: FAM120A, EphA2 (Cat. No. 12927, Cell Signaling Technologies), Phospho-EphA2 Ser897 (Cat. No. 6347, Cell signaling Technologies), Phospho-AKT Ser473 (Cat. No. 4060, Cell Signaling Technologies), Phospho-ERK1/2 (Cat. No. 9101, Cell signaling Technologies), and β-actin (Cat. No. sc-47778). The following secondary antibodies from Santa Cruz were used: anti-rabbit HRP-linked (Cat. No. sc-2357) and anti-mouse HRP-linked (Cat. No. sc-2357). Immunoblot quantification was performed using ImageJ.

### Immunofluorescence and confocal microscopy analysis

For Immunofluorescence analysis, cells were seeded in Ibidi plates (Fischer Scientific) in RPMI containing 10% FBS and 1% P/S and incubated overnight. Cells were then serum-starved for 24 hours and washed with PBS, fixed with 4% paraformaldehyde in PBS for 20 minutes at RT. Subsequently, cells were permeabilized in 0.2% Triton X100 in PBS for 10 minutes. Cells were then blocked with 5% BSA in 1X PBS for 30 minutes at RT and incubated with the following primary antibodies: FAM120A (Cat. No. 21529-1-AP, Protein-tech), EphA2 (Cat. No. 12927**,** Cell Signaling Technologies), Phalloidin (Cat. No. ab176753, Abcam) in 1% BSA overnight at 4 °C or 1 hour at RT. Later, cells were incubated with goat anti-rabbit IgG (H + L) Alexa Fluor™ Plus 594 (Thermo Fisher Scientific) and goat anti-Mouse Alexa Fluor™ Plus 488 (Thermo Fisher Scientific) conjugated secondary antibodies for 1 hour at RT. Cells were also stained with DAPI (Thermo Fisher Scientific) at 300nM. Cells were visualized with an Olympus IX81 fluorescent microscope (Olympus, Tokyo, Japan) equipped with a Retiga 6000 camera (QImaging, Surrey, Canada) or Olympus FV3000 Scanning Confocal Microscope. Immunofluorescence co-localization analysis was performed using the ImageJ program and is expressed as Pearson’s coefficient. F-actin filaments per cell were quantified using the ImageJ program.

### RhoA pull-down activation assay

RhoA activation assays were performed following the manufacturer’s instructions (Cat. #BK036, Cytoskeleton Inc.). Briefly, Serum-starved cells were stimulated with progranulin, and lysates were collected. 1 mg of lysates was precipitated with rhotekin-beads for 1 h at 4°C. Beads were then washed with wash buffer, resuspended in 2X Laemmli buffer, and loaded on a 12% polyacrylamide gel.

### Proximity Ligation Assay (PLA)

PLA was performed using the Duolink Proximity Ligation Assay according to the manufacturer’s instructions (Sigma-Aldrich). Cells were incubated with anti-FAM120A (Cat. No. 21529-1-AP, Protein-tech) and anti-EphA2 antibodies (Cat. No. 12927**,** Cell Signaling Technologies) ON at 4°C. Negative control was performed by incubating with only one primary antibody ON at 4°C. Images were taken as described above for immunofluorescence analysis.

### Immunohistochemistry (IHC) analysis

FAM120A and EphA2 expression levels were analyzed by IHC on a BioMax BC tissue microarray (TMA) (Cat. No. BL1501a) at the Translational Core Facility of the Kimmel Cancer Center, Thomas Jefferson University. The TMA has 8 benign bladder tissue samples, 2 urothelial carcinoma adjacent tissue samples, and 140 malignant urothelial carcinoma tissue samples consisting of squamous cell, mucinous adenocarcinoma, endocrine, intestinal adenocarcinoma, and low- or high-grade invasive urothelial carcinoma. Out of those 140 malignant urothelial carcinomas, there were 40 T1, 58 T2, 40 T3, and 2 T4 stage tissue samples. The anti-FAM120A monoclonal antibody (Proteintech) or anti-EphA2 monoclonal antibody (Santa Cruz) was used at a 1:200 dilution. Detailed specifications of the bladder tissue array can be found at: https://www.tissuearray.com/tissue-arrays/Bladder/BL1501a. Quantification of the FAM120A or EphA2 staining in urothelial carcinoma tissues was performed using ImageScope software from Leica.

### Gene depletion

To generate T24, 5637, and UMUC-3 cell lines with a stable depletion of FAM120A, we infected cells with a retroviral plasmid expressing either shRNAs specific for FAM120A (Millipore Sigma) or oligo controls expressed in pLKO.1 - TRC cloning vector was a gift from David Root (Addgene plasmid # 10878; http://n2t.net/addgene:10878; RRID: Addgene_10878). To generate viral particles, HEK-293FT cells were transfected with VSV-G envelope plasmid, GAG-POL packaging plasmid, and the shRNAs for either FAM120A or scrambled RNA sequence using a Calcium Phosphate Transfection Kit (Milipore Sigma). HEK-293FT-transfected conditioned media supplemented with 8 µg/ml polybrene (Sigma-Aldrich) was used to transduce cells as described [24, 25].

Target sequences of the two FAM120A-specific shRNA plasmids were:

o 5”GCTTCCTTTCACTGGAGTTTA3”
o 5”GCCTTGAATAATGACTCTAAA3”

Selection was done in medium supplemented with 2 μg/ml of puromycin. After selection, pools of FAM120A-depleted T24, 5637, and UMUC-3 cells were tested for FAM120A expression levels by western immunoblot using anti-FAM120A antibody (Cat. No. A303-889A, Fortis Life Sciences).

### Wound healing assay

Cells were seeded onto 6-well plates in complete media and the next day transferred into SFM for 24 h. We generated a wound using a p200 pipette tip, and then cells were washed twice with PBS 1X. At that time, SFM with or without progranulin was added. Images of the wound were taken at the indicated time points using a Dmi1 inverted microscope (Leica, Wetzlar, Germany). Wound area was measured using the ImageJ program.

### Migration and invasion assay

Cell migration was performed using transwell migration assays with 8.0 µm pore polycarbonate membrane inserts (Corning, Glendale, AZ, USA). Serum-starved cells were seeded onto the upper chamber in SFM at a cell density of 5,000 cells. The lower chamber was filled with 750 µL of SFM with or without 100 nM progranulin. After 18 h, cells on the filter upper surfaces were removed with a cotton swab, while cells on the filter lower surfaces were fixed with 100% ice-cold methanol, stained with 0.5% crystal violet, and counted using a Dmi1 inverted microscope (Leica, Wetzlar, Germany).

Cell invasion through a three-dimensional extracellular matrix was measured using inserts containing an 8 µm pore-size membrane with a uniform layer of Matrigel matrix (Corning). Matrigel-coated inserts filled with SFM were pre-incubated at 37 °C for 2 hours.

### Cell cytotoxicity assay

Three thousand (T24) or 5000 (UMUC-3 or 5637) cells were seeded into 96-multiwells flat bottom plate (CellTreat) in media supplemented with 10% FBS. The next day, cells were transferred to 1% FBS-supplemented media with or without cisplatin (Sigma-Aldrich, St Louis, MO, USA) at the concentrations of 1,10, 20, 30, and 40 μM. After 24 h. Cells were fixed with 10% trichloroacetic acid at 4 °C for 2 h and stained with 0.4% sulforhodamine B (Thermofischer) in 1% acetic acid. The absorbance was measured at 565 nm using a SpectraMax iD3 (Molecular Devices).

### Clonogenic assay

Three hundred cells were seeded into 6-well plates and grown in complete media. After 10 −15 days, cells were washed with PBS 1X, fixed with 100% ice-cold methanol, and stained with 0.5% crystal violet solution and photographed. Colonies were counted using the ImageJ program.

### Soft agar colony formation assay

Colony formation in soft agar was performed as previously described [14, 16]. Briefly, 3000 cells were seeded into a 0.2% agar layer in a 35 mm cell dish. After 3 weeks, colonies >150 μm were counted under the microscope.

### 3D Spheroid formation assay

3D spheroid assays were performed using the single spheroid formation protocol as described [26]. Briefly, 10,000 cells were resuspended in 3D experimental media described in [26] and seeded in U-shaped ultra-low attachment multiple well plates (Corning). Cells were centrifuged at 340× *g* for 30 min and incubated at 37 °C. Spheroids were monitored on a daily basis and photographed using a Dmi1 inverted microscope (Leica, Wetzlar, Germany).

### In vivo xenograft

3-month-old male Nu/Nu mice (strain code 088) were purchased from Charles River Laboratories (Wilmington, MA) and housed at the Thomas Jefferson University (TJU) animal research facility in sterilized filter-top cages under a 12-h light-dark cycle, humidity- and temperature-controlled conditions. All animal procedures were approved by the Animal Care and Use Committee of TJU. 1x 10^6^ cells/100 µl PBS were ectopically implanted into the right posterior flank of mice using a sterile 20-gauge needle. 8 mice were injected with shCTR- and 8 with FAM120A-depleted UMUC-3 cells. Throughout the study, the health status of the mice was checked every 2 days. After Day 10 post-injection, when manual palpation revealed the existence of a tumor around the injection site, tumor sizes were assessed throughout the experiment by caliper measurements of length (L) and width (W), and tumor volume (V) was calculated using the formula: V (mm^3^) = 0.5 x L x W^2^. When the largest tumor reached 2000 mm^3^ in size, all mice were sacrificed by CO_2_ inhalation and tumors were surgically dissected and remeasured extracorporeally to ensure accuracy of *in vivo* measurement.

### Quantification and statistical analysis

Data are presented as mean ± SD from at least three independent experiments. Statistical analyses were performed using GraphPad Prism (version 10.4.1). Comparisons between groups were made using one-way ANOVA or unpaired *t*-test, and *p* < 0.05 was considered statistically significant.

## Results

### Proteomic approaches to characterize the interactome of progranulin-activated EphA2

**Fig. 1:**
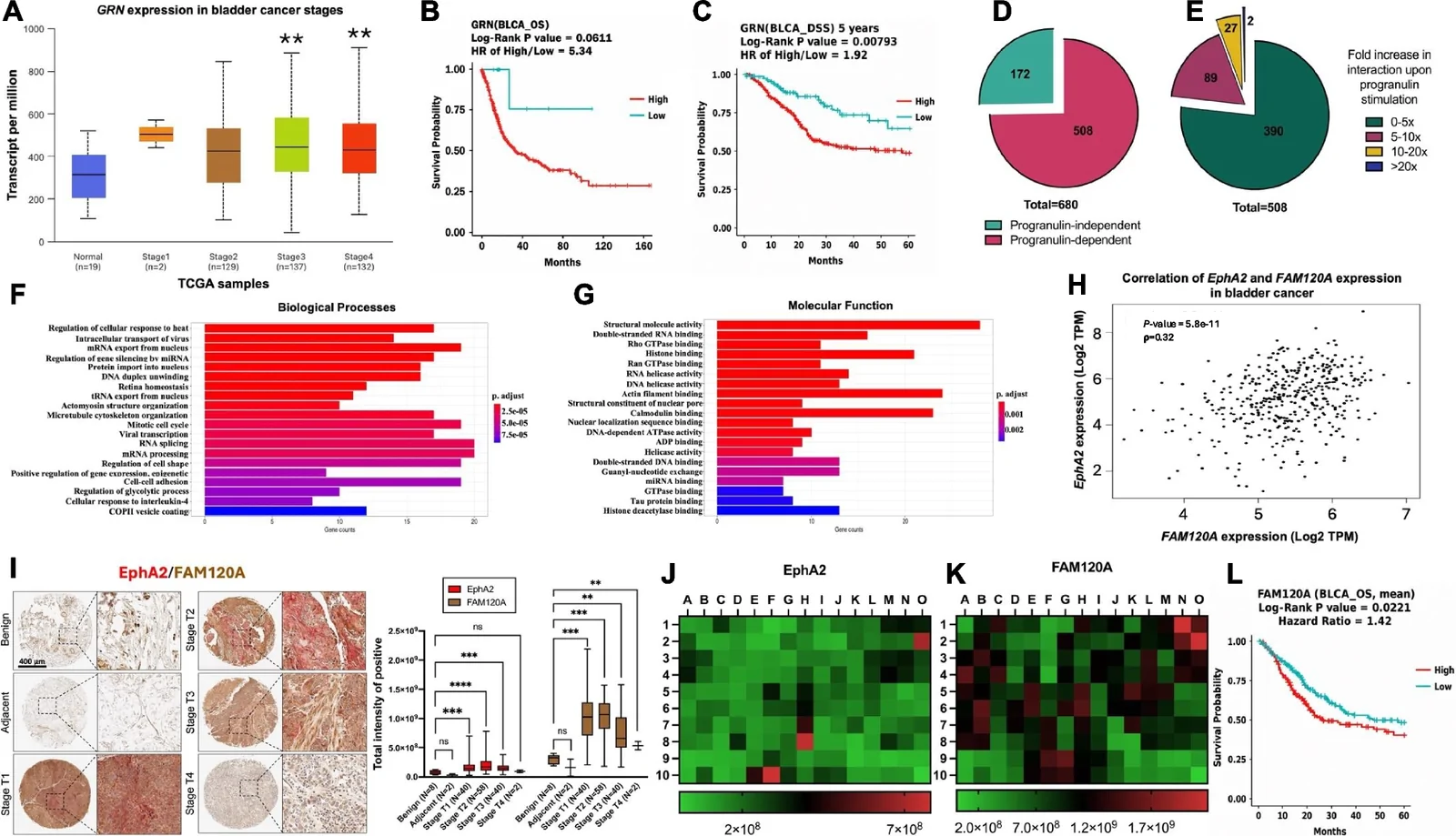
Proteomic approaches to characterize the interactome of progranulin-activated EphA2 and FAM120A expression levels in BC. **A** Assessment of *GRN* mRNA expression levels in various BC tissues using the UALCAN database. **B** Kaplan-Meier analysis of overall survival (OS) or **C** disease-specific survival (DSS) in bladder cancer (BLCA) patients stratified by *GRN* expression. **D** Classification of identified EphA2 interactors divided into progranulin-dependent or progranulin-independent binding. **E** Classification of progranulin-dependent EphA2 interactors based on fold increase in binding. **F-G)** EphA2 interactors’ role in various biological processes and molecular function as analyzed by interrogating The Gene Ontology Resource. **H** Correlation of *EphA2* and *FAM120A* expression in BC is measured in Spearman’s correlation coefficient using the GEPIA2 platform. **I** Human BC TMAs with 75 cases of validated cases of various types, stages, and grades of BC and normal and adjacent tissue controls. EphA2 (Red) and FAM120A (Brown) are expressed at high levels in all stages compared to normal and adjacent tissue controls. Selected regions are magnified (5X) in boxes. Quantification of EphA2 and FAM120A staining levels was performed using the ImageScope program. All cases in the TMAs were analyzed, quantified, and plotted using GraphPad Prism software Version 10.4.1. Unpaired *t*-tests or one-way ANOVA were run to determine the statistical significance of each class of cancer compared to normal bladder and adjacent tissues. **J-K** Heatmaps representing the total positive intensity of EphA2 or FAM120A in BC TMAs. **L** Kaplan-Meier analysis of OS in BLCA patients stratified by *FAM120A* expression. High *FAM120A* expression was associated with significantly reduced overall survival compared with low expression (log-rank M1*P =* 0.0221; HR = 1.42). ns= not significant, *p < 0.05, **p < 0.01, ***p < 0.001 ****, *p* < 0.0001

We have previously shown that the progranulin/EphA2 axis has a pro-tumorigenic action in BC and may serve as a novel biomarker for BC progression [3, 17–19, 27]. We previously demonstrated by IHC analysis that progranulin and EphA2 protein levels are upregulated in BC tissues compared to normal controls [13, 19]. To further define the clinical relevance of progranulin (*GRN)* in BC, we analyzed transcriptomic data from The Cancer Genome Atlas (TCGA) BC cohort. *GRN* expression varied across tumor stages and was significantly elevated in Stage III and Stage IV tumors compared to normal bladder tissues, suggesting that increased progranulin expression is associated with advanced disease (Fig. 1A). Next, patients with high *GRN* expression exhibited a trend toward poorer overall or disease-specific survival compared with those expressing low levels of *GRN* (Fig. 1B, C). However, the molecular mechanism of progranulin/EphA2-dependent oncogenic signaling in BC is still poorly understood. Thus, we used proteomic approaches to characterize the progranulin-dependent EphA2 interactome in BC using T24 cells exposed to soluble progranulin (100 nM) [19]. Considering the spectral count of control-C1 and PGRN-P1, we identified a total of 680 EphA2 interactors, 172 of which were progranulin-independent and 508 were progranulin-dependent interactors (Fig. 1D). Furthermore, we conducted a sub-analysis to assess the fold increase in binding between EphA2 and its interactors upon exposure to soluble progranulin. We found a 0–5-fold increase in 390, 5–10-fold in 89, 10-20-fold in 27, and>20-fold in 2 progranulin-dependent EphA2 interactors (Fig. 1E). Within the set of progranulin-dependent EphA2 interactors, FAM120A emerged as a particularly appealing candidate because its interaction with EphA2 was markedly enhanced following progranulin stimulation by 6-fold. In addition, FAM120A has been previously characterized as a scaffold protein involved in oncogenic and receptor tyrosine kinase-associated signaling pathways, including AKT-mediated survival and migration. Given the established roles of progranulin and EphA2 in promoting aggressive BC phenotypes, these findings led us to hypothesize that FAM120A may function as a critical mediator of progranulin-induced EphA2 signaling and downstream tumor-promoting activities.

Next, we analyzed the role of identified EphA2 interactors in biological processes and molecular functions using the Gene Ontology resource (https://geneontology.org) (Fig. 1F, G). In biological processes, we discovered the greatest number of identified EphA2 interactors involved in mRNA export from the nucleus, regulation of gene silencing by miRNA, protein import into the nucleus, etc. (Fig. 1F), whereas in molecular functions, we found most of the identified EphA2 interactors having a role in Rho GTPase binding, Ran GTPase binding, actin filament binding, etc. (Fig. 1F).

### EphA2 and FAM120A expression is upregulated and correlated in BC

While FAM120A action as a multi-functional adaptor/scaffold protein involved in signal transduction has been previously demonstrated [21, 22, 28, 29], there are no data suggesting either an interaction with EphA2 or a role in BC. Thus, to quantify the relationship between *EphA2* and *FAM120A* expression in BC, we first performed a correlation analysis (Fig. 1H) using the TCGA-BC cohort through the GEPIA2 platform (http://gepia2.cancer-pku.cn/#correlation). Spearman correlation analysis demonstrated a significant positive association between *EphA2* and *FAM120A* mRNA expression (Spearman's ρ = 0.32, *P =* 5.8 × 10⁻^11^), indicating that tumors expressing higher levels of *FAM120A* also tend to express higher levels of *EphA2*. we performed IHC to evaluate EphA2 and FAM120A protein expression levels on a commercially available BC TMA containing a total of 75 cases of BC. We demonstrated that EphA2 and FAM120A protein expression was significantly upregulated in BC tissues compared to normal or benign bladder tissue controls (Fig.1I). To further evaluate the relationship between EphA2 and FAM120A expression, protein expression levels were visualized across individual patient samples using heatmap analysis (Fig. 1J, K). EphA2 expression was relatively low and uniform in benign and adjacent tissues but was elevated in a subset of tumor specimens, particularly in higher-stage tumors. FAM120A expression exhibited a similar pattern, with increased expression observed predominantly in tumor samples compared with non-malignant controls. Notably, several specimens displaying high EphA2 expression also exhibited elevated FAM120A levels, suggesting a positive association between the two proteins in BC tissues. These data support the existence of a coordinated EphA2-FAM120A signaling axis that may contribute to BC.

To investigate the clinical relevance of *FAM120A* expression in BC, we performed Kaplan-Meier overall survival analysis using the DriverDB database [30] (Fig. 1L). Patients with high *FAM120A* expression exhibited significantly poorer overall survival compared with those expressing low levels of *FAM120A*. Furthermore, the hazard ratio was 1.42, indicating that patients with high *FAM120A* expression had an approximately 42% greater risk of death than patients with low *FAM120A* expression. These findings suggest that FAM120A overexpression is associated with unfavorable clinical outcomes in BC and support a potential oncogenic role for FAM120A in BC. Collectively, the data identify FAM120A as a candidate prognostic biomarker whose elevated expression correlates with poorer patient survival.

**Fig. 2:**
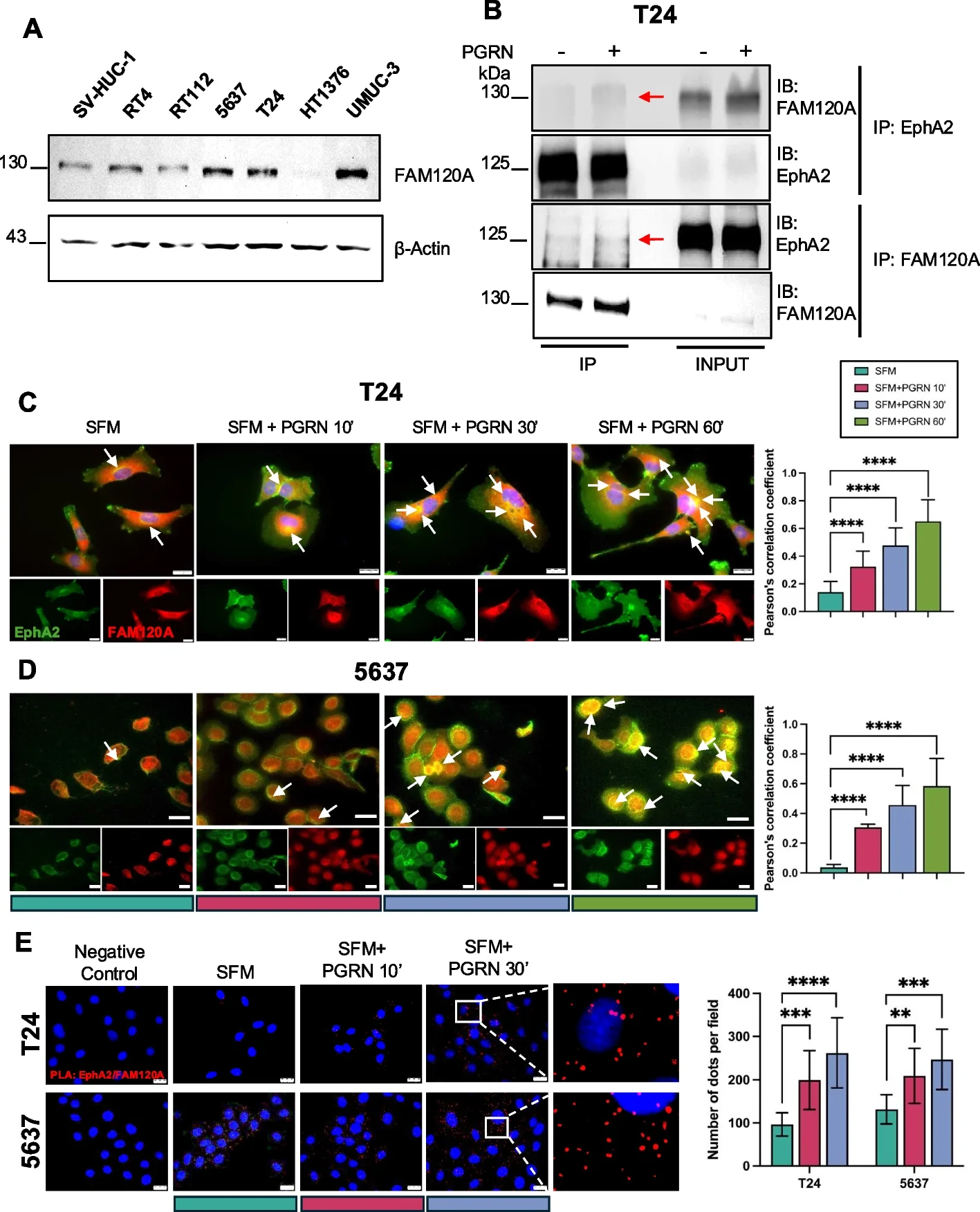
EphA2 and FAM120A interaction is progranulin-dependent in BC cells. **A** FAM120A protein levels were analyzed by immunoblot in cell lysates from SV-HUC-1, RT4, RT112, 5637, T24, HT1376, and UMUC-3 cells. **B** Co-immunoprecipitation analysis was performed as described in Materials and Methods. T24 cells were serum-starved for 24 hours and then stimulated with or without progranulin at 100 nM for 15 minutes. Lysates (5 mg) were immunoprecipitated with anti-EphA2 or anti-FAM120A antibodies. Proteins were detected by immunoblot with specific anti-EphA2 or anti-FAM120A antibodies. 30 µg of total lysates were run as inputs. The arrow points to either the EphA2 or FAM120A band. **C-D** Cells were seeded on Ibidi plates, 20000 cells/chamber. The next day, cells were starved for 4 hours in SFM described in Materials and Methods. Cells were stimulated with or without progranulin 100nM at the indicated time points and were then analyzed for EphA2 and FAM120A expression by immunofluorescence. Cell nuclei were stained with DAPI. Bars = 20 µm. Insets = higher magnification of the relative areas within the white boxes. Quantification of colocalization is expressed as Pearson’s Coefficient, representing the fraction of red (FAM120A) overlapping with green (EphA2). Figures are representative of three independent experiments and 15 images per experiment. **E** T24 and 5637 cells were serum-starved for 24 hours, and colocalization of EphA2 and FAM120A was analyzed by PLA upon progranulin stimulation for the indicated time points. Bars = 20 µm. T24 and 5637 cells probed with a single primary antibody were used as negative controls. Cell nuclei were stained with DAPI. Insets = higher magnification of the relative areas within the white boxes. Quantification represents the number of dots per field. Statistical significance was determined using GraphPad Prism version 10.4.1. *p < 0.05, **p < 0.01, ***p < 0.001, ****p < 0.0001

Next, we assessed FAM120A levels by immunoblotting in various BC cell lines (Fig.2A) and showed that FAM120A protein was well-expressed in all BC cell lines, with the only exception of HT1376 cells. Notably, FAM120A was upregulated in 5637, T24, and UMUC3 invasive BC cells as compared to SV-HUC-1, which was used as a control, being derived from immortalized human normal urothelium (Fig. 2A).

### EphA2-FAM120A interaction is progranulin-dependent in BC cells

To corroborate the proteomic data and confirm the EphA2/FAM120A interaction, we conducted co-immunoprecipitation experiments in T24 cells, the cells we used for the proteomic analysis, which express high levels of endogenous FAM120A protein (Fig. 2A). T24 cells were serum-starved for 24 hours and supplemented with or without progranulin (100 nM) for 15 minutes. We were able to co-precipitate endogenous EphA2 and FAM120A using either anti-EphA2 or anti-FAM120A antibodies (Fig. 2B). Notably, EphA2/FAM120A interaction was enhanced upon progranulin stimulation (Fig. 2B).

We then performed immunofluorescence analysis to assess the colocalization of EphA2 and FAM120A in T24 and 5637 cells and demonstrated significant colocalization (*p*<0.0001) between EphA2 and FAM120A in both cell lines. Remarkably, these interactions were significantly enhanced upon progranulin stimulation in a time-dependent manner (Fig. 2C, D).

To further confirm EphA2 and FAM120A functional interaction, we tested EphA2 and FAM120A co-localization by PLA in T24 or 5637 cells (Fig. 2E). Notably, we found that EphA2-FAM120A colocalized in both cell lines and this interaction was significantly enhanced upon progranulin stimulation as compared to non-treated control cells (SFM) (Fig. 2E). Thus, these results strongly demonstrate that EphA2 and FAM120A are in close proximity (30–40 nm apart), thereby confirming their interaction in BC cells [31].

### FAM120A modulates progranulin-dependent activation of the AKT and ERK1/2 pathways

**Fig. 3:**
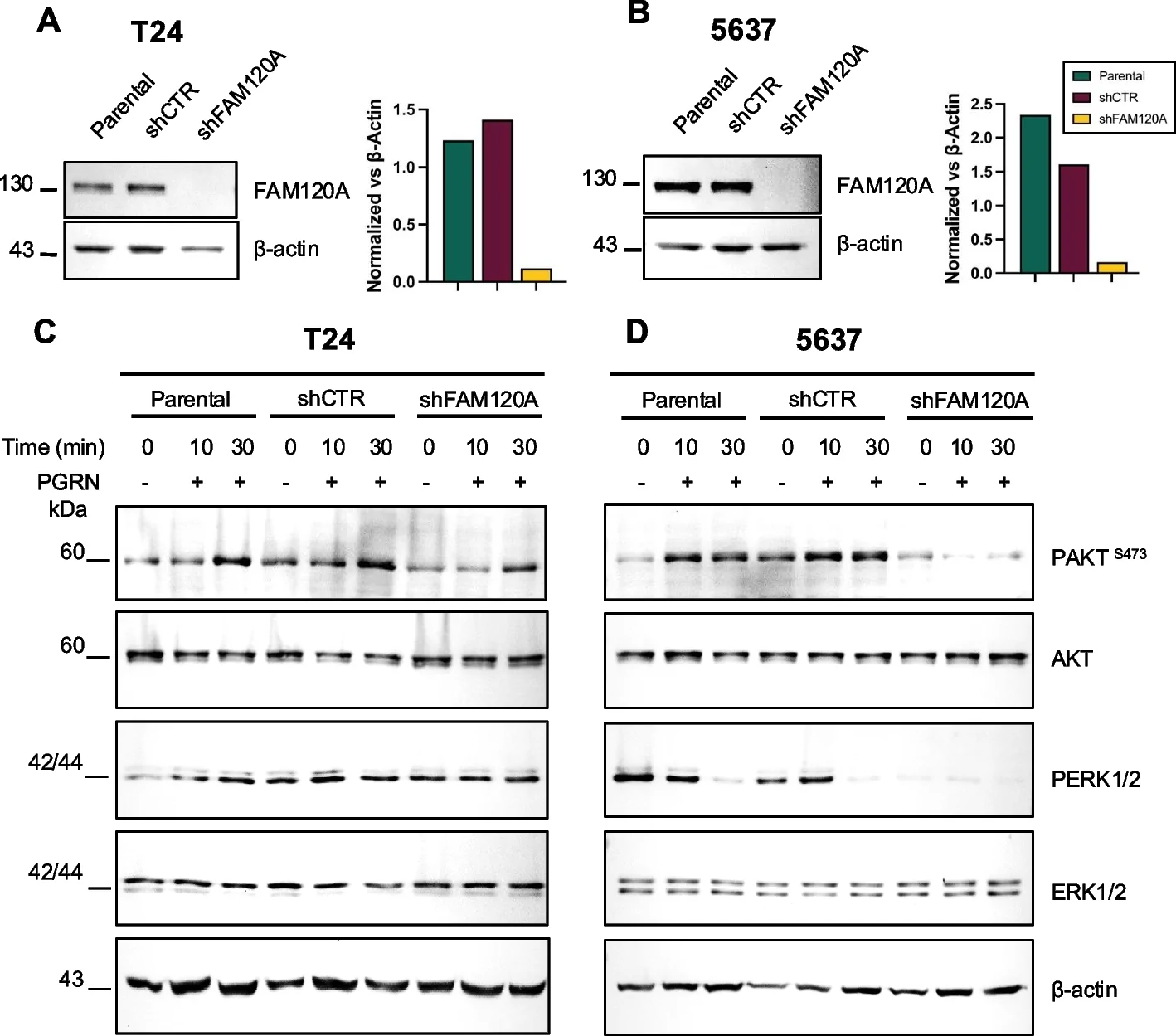
FAM120A colocalizes with EphA2 and modulates progranulin-dependent activation of the AKT and ERK1/2 pathways. **A-B** Immunoblot confirming FAM120A depletion in T24 and 5637 cells. Quantification was performed using the ImageJ program. **C-D** Parental, shCTR- and FAM120A-depleted T24 and 5637 cells were serum-starved for 24 h and stimulated with or without 100 nM progranulin for the indicated time points. Levels of total and phosphorylated AKT and ERK1/2 were assessed by immunoblot. All the experiments were performed at least two times independently

To characterize the functional relevance of the EphA2 and FAM120A interaction and the role of FAM120A in modulating progranulin-mediated biological responses, we generated control (shCTR) and FAM120A-depleted T24 and 5637 cells using lentiviral shRNA approaches and achieved a substantial depletion of endogenous FAM120A protein in both cell lines (Fig. 3A, B). Because we previously demonstrated that progranulin-mediated activation of the AKT and ERK1/2 pathways is required for its oncogenic responses in BC [17, 19, 32], we initially tested progranulin-induced AKT and ERK1/2 activation by immunoblot in T24 and 5637 cells, comparing parental, control, and FAM120A-depleted cells.

We found that FAM120A depletion caused a significant inhibition of progranulin-induced AKT activity in both cell lines (Fig. 3C, D). In contrast, ERK1/2 activation was significantly blunted in 5637 cells but not in T24 cells (Fig. 3C, D), suggesting that FAM120A depletion might have slightly different effects depending on cell context. However, these results support a critical role of FAM120A in modulating progranulin/EphA2 downstream signaling.

### FAM120A regulates progranulin-dependent migration and invasion of BC cells

**Fig. 4:**
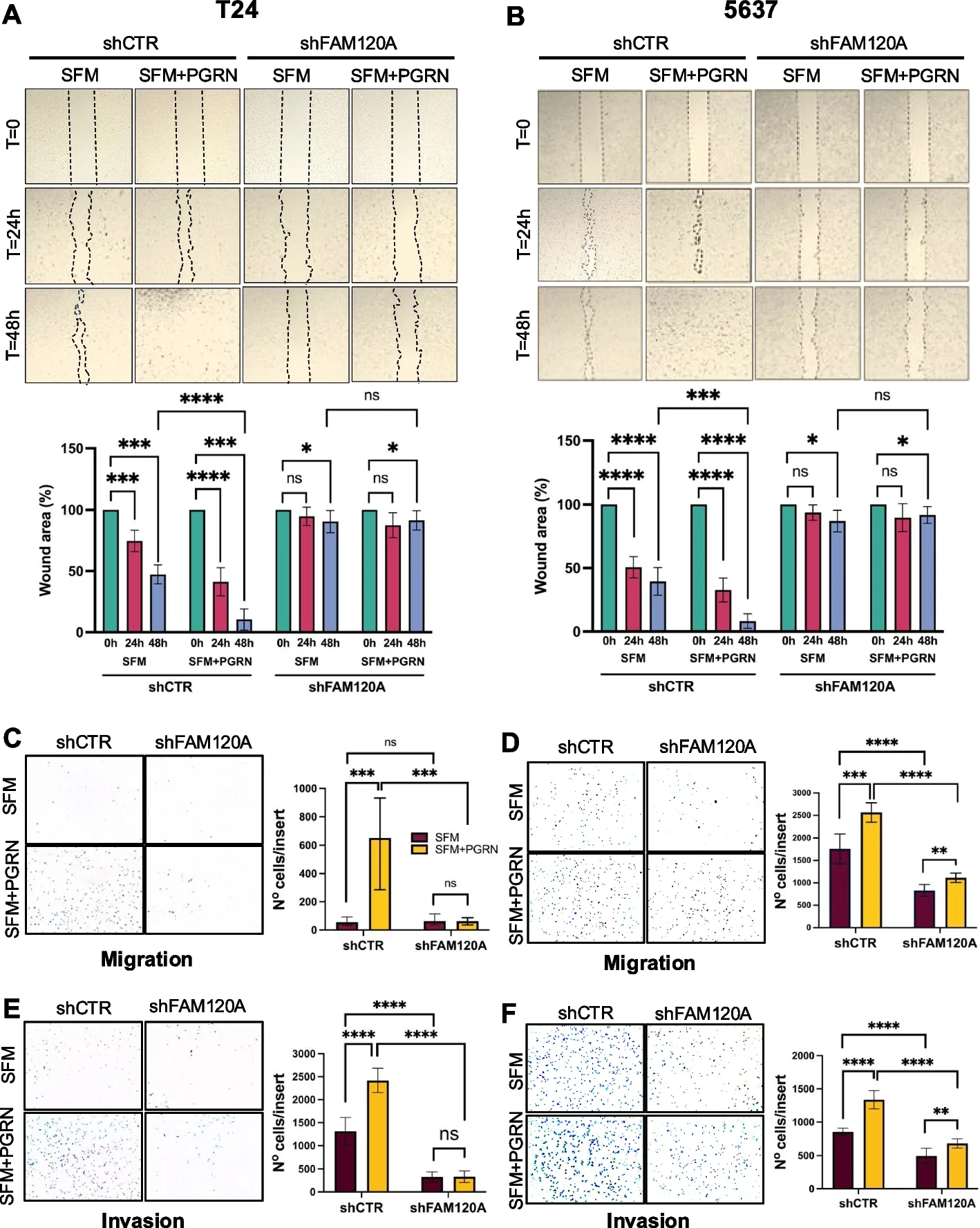
FAM120A regulates progranulin-dependent migration and invasion of BC cells. **A-B** Lateral motility was measured using wound healing assays. shCTR or FAM120A-depleted T24 or 5637 cells were serum-starved for 24 h, and a wound was generated using a p200 tip. Cells were incubated with SFM or SFM+PGRN (progranulin) (100nM). Wound closure was monitored at the indicated time points with a DMi1 inverted microscope. **C-D** Migration of shCTR- and FAM120A-depleted T24 and 5637 cells was assessed using transwells. The bottom chamber was filled with SFM or SFM + PGRN (100 nM), and cells were allowed to migrate for 18 h at 37 °C. **E-F** Invasion of shCTR- and FAM120A-depleted T24 and 5637 cells was assessed using Matrigel-coated transwells. The bottom chamber was filled with SFM or SFM + PGRN (100 nM). Cells were allowed to invade for 24 h at 37 °C. Data are the average of at least three independent experiments run in duplicate. ns= not significant, *p < 0.05, **p < 0.01, ***p < 0.001 ****, *p* < 0.0001

We previously showed that progranulin-depleted BC cells showed considerable inhibition in their capacity to close a wound [15]. Thus, we performed wound healing assays to determine whether FAM120A may modulate the lateral motility of BC cells. While control-infected T24 and 5637 cells significantly closed the wound upon progranulin stimulation, FAM120A-depleted cells were significantly inhibited in their lateral motility capacity (Fig. 4A, B).

We previously demonstrated that the progranulin/EphA2 axis is a critical mediator of cell motility and invasion in several cancer models, including bladder and prostate cancer and mesothelioma [3, 12–19, 33–36]. Thus, we determined whether FAM120A plays any role in modulating BC cell motility and invasion by comparing the ability of FAM120A-depleted cells to migrate and invade through Matrigel. FAM120A-depleted T24 and 5637 cells showed a significant reduction in progranulin-dependent cell motility as assessed by migration assays in boyden chambers when compared to controls in both T24 and 5637 cells (Fig. 4C, D). In 5636 cells, FAM120A depletion also affected basal cell motility, as compared to control-infected cell lines, indicating that in this specific cell model, FAM120A may also play a more general role in regulating cell migration. Accordingly, FAM120A depletion considerably reduced the ability of both T24 and 5637 cells to invade through Matrigel-coated chambers (Fig. 4E, F), indicating that FAM120A expression is critical for progranulin-dependent motility and invasion of BC cell lines.

### FAM120A is required for clonogenic and 3D spheroid formation/growth of BC cells

**Fig. 5:**
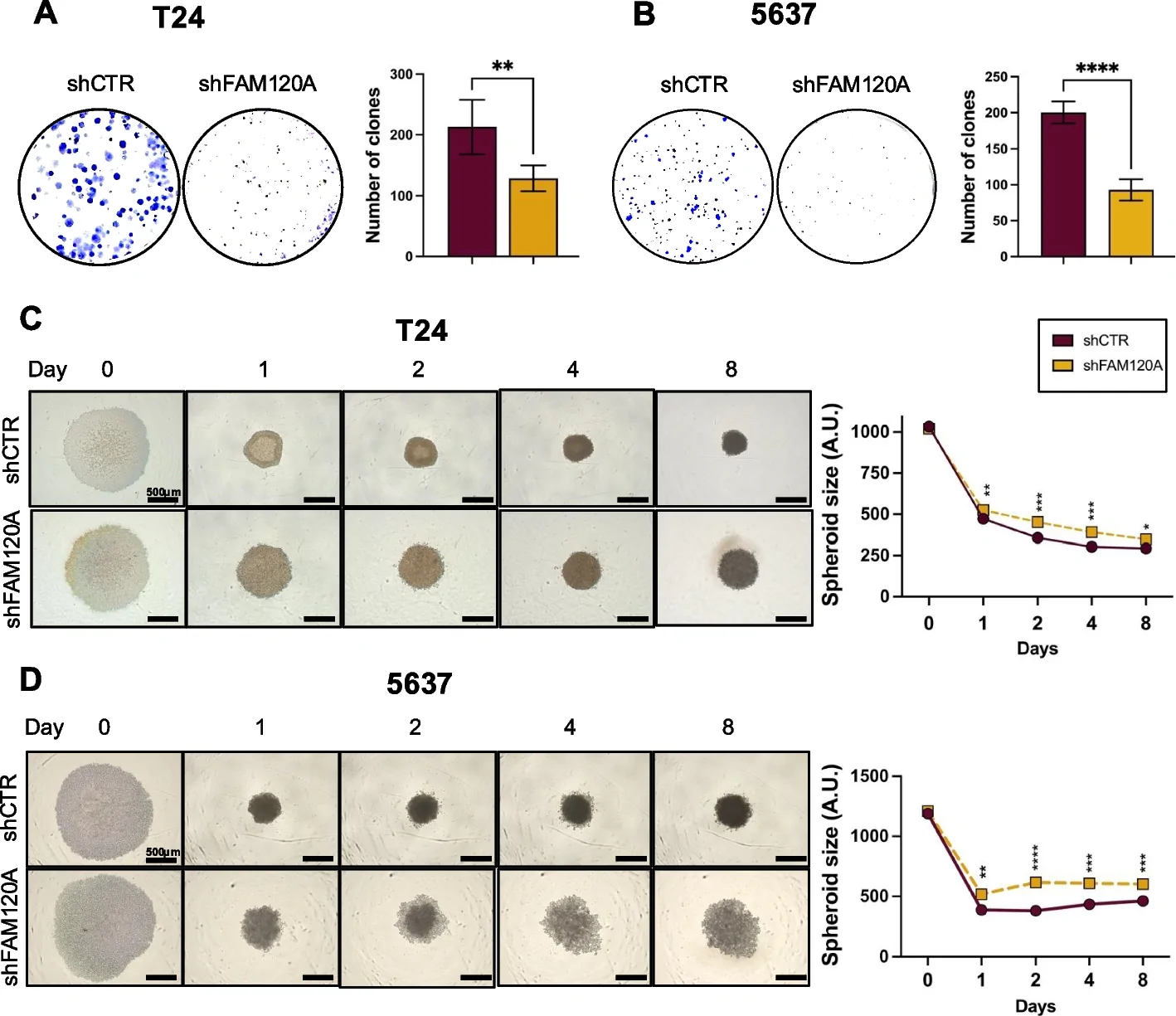
FAM120A is required for clonogenic and 3D spheroid formation/growth capacities. **A-B** Monolayer clonogenic assays were performed in T24 and 5637 cells. The data shown are the average of 3 independent experiments performed in duplicate. **C-D** Spheroid formation assay was performed in T24 and 5637 as described in Materials and Methods and monitored at the indicated time points with a DMi1 inverted microscope. Spheroid size was measured using the ImageJ program. The data shown are the average of two experiments performed in at least 10 replicates. A.U.=Arbitrary Unit. ns= not significant, *p < 0.05, **p < 0.01, ***p < 0.001 ****p < 0.0001

Next, we evaluated the ability of FAM120A to regulate clonogenicity in monolayer T24 and 5637 cultures. We discovered that FAM120A depletion severely decreased the ability of both T24 and 5637 cells to form clones in monolayer growth as compared to control-infected cells (Fig. 5A, B), suggesting that loss of FAM120A impairs long-term growth in monolayer of BC cells.

We further corroborated these results by generating FAM120A-depleted 5637 cells using a second shFAM120A-expressing lentiviral vector. This vector significantly reduced FAM120A expression levels as compared to parental and control-infected 5637 cells (Supplemental Fig. 1 A) with a strong inhibition of migration, invasion, and clonogenicity of 5637 cells (Supplemental Fig. 1B, C, D). These data confirmed the above results and further indicated that FAM120A is essential for mediating progranulin-induced migratory and invasive abilities of BC cells.

3D spheroids are currently considered an important preclinical experimental model for assessing oncogenic phenotypes of cancer cells [26]. Thus, we assessed the ability of FAM120A-depleted T24 or 5637 to form a 3D spheroid in suspension using a well-established protocol from our laboratories [26]. Notably, FAM120A-depleted T24 cells demonstrated slower spherogenesis compared to shCTR/T24 cells (Fig. 5C), while FAM120A-depleted 5637 cells showed a severe inhibition of spherogenesis as compared to shCTR/5637 cells (Fig. 5D), suggesting that FAM120A contributes to the maintenance of tumor cell aggregation, viability, and potentially stem-like properties, reinforcing its role in promoting tumorigenic potential and 3D growth capacity in BC cells.

### FAM120A regulates progranulin-induced F-actin network remodeling

**Fig. 6:**
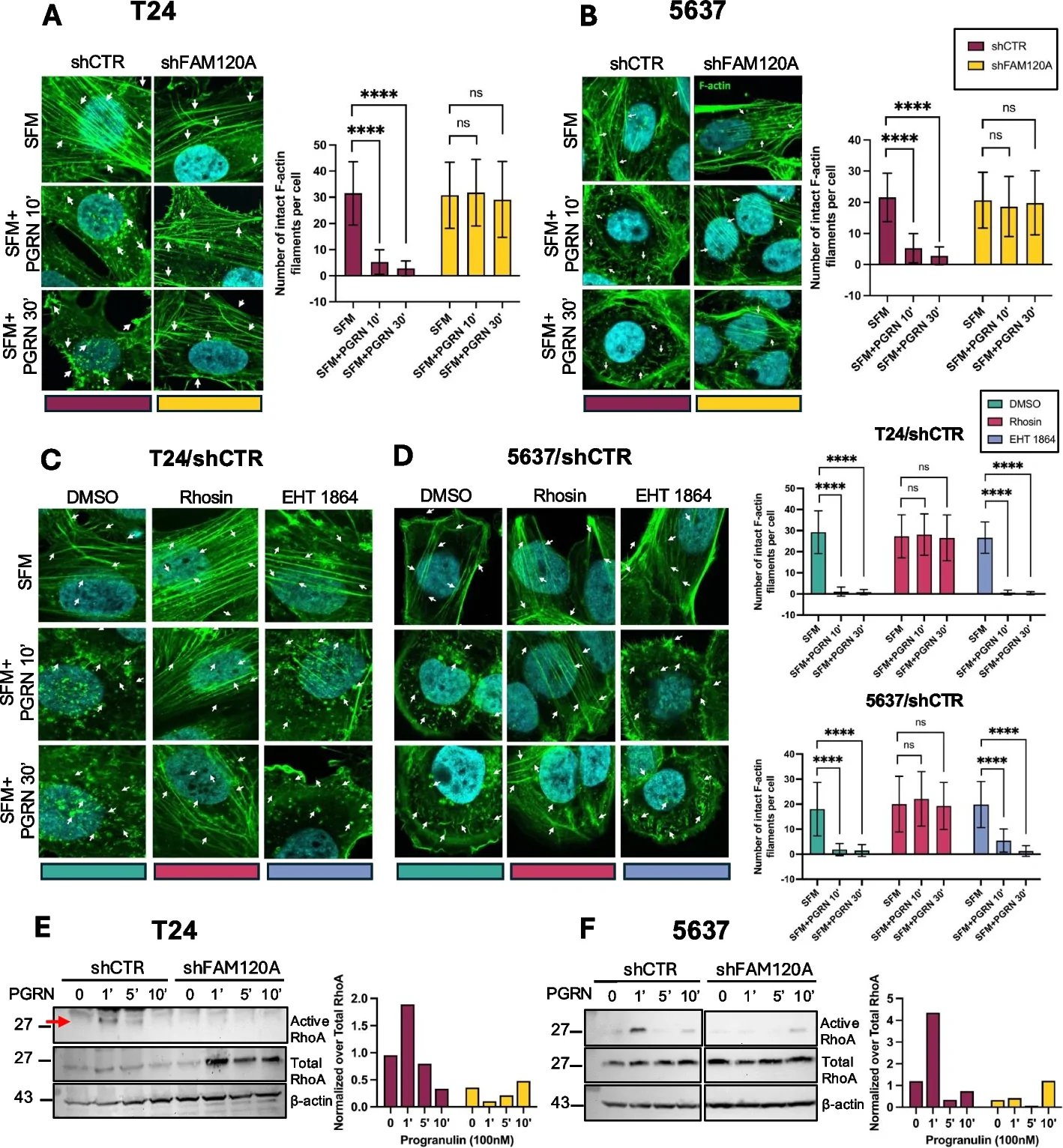
FAM120A regulates progranulin-induced actin network remodeling. T24 and 5637 cells were seeded on Ibidi plates, 20000 cells/chamber. The next day, cells were starved in SFM for 24 h. **A-B** Cells were stimulated with or without progranulin 100 nM at the indicated time points, stained for F-actin using anti-phalloidin antibodies, and analyzed by immunofluorescence. Cell nuclei were stained with DAPI. Arrows point to F-actin filament remodeling. **C-D** Cells were pre-treated with Rhosin 30 mM or EHT 1864 50 mM for 30 minutes, followed by stimulation with progranulin 100 nM at the indicated time points, stained for F-actin using anti-phalloidin antibodies, and assessed by immunofluorescence. Cell nuclei were stained with DAPI. Arrows point to intact F-actin or F-actin filament remodeling. Figures are representative of two independent experiments and 15 images per experiment. Intact F-actin filaments were counted using ImageJ. **E-F** shCTR- and shFAM120A-depleted T24 or 5637 cells were serum-starved for 24 h and stimulated with progranulin for the indicated time points. Active RhoA (GTP-bound RhoA) was pulled down using rhotekin beads, and the levels of active RhoA were measured using immunoblotting. Levels of Total RhoA were measured in whole lysates before the pull-down assay. Quantification was performed using the ImageJ program. ns= not significant, *p < 0.05, **p < 0.01, ***p < 0.001 ****p < 0.0001

To mechanistically define the role of FAM120A in progranulin-mediated motility of BC cells, we focused on its potential involvement in cytoskeletal remodeling, a process essential for cell motility [37, 38]. This rationale is also based on our previous studies, where we demonstrated that progranulin, by interacting with the F-actin binding protein drebrin, plays a critical role in modulating F-actin cytoskeletal remodeling of BC cells [14]. Accordingly, we performed F-actin staining of T24 and 5637 cells to assess whether FAM120A contributes to modulating progranulin-dependent F-actin cytoskeletal rearrangements. Under SFM conditions, both shCTR and FAM120A-depleted T24 and 5637 cells showed well-organized F-actin fibers, indicating an intact cytoskeletal architecture (Fig. 6A, B). In contrast, upon progranulin stimulation, both shCTR T24 and 5637 cells demonstrated marked F-actin disassembly accompanied by the formation of cortical actin structures, consistent with dynamic cytoskeletal remodeling associated with increased motility (Fig. 6A, B). In contrast, FAM120A-depleted T24 or 5637 cells maintained prominent and organized F-actin filaments, showing limited cytoskeletal rearrangement in response to progranulin (Fig. 6A, B). These findings suggest that FAM120A is essential for progranulin-induced F-actin reorganization, likely acting as a downstream effector that facilitates the cytoskeletal plasticity required for enhanced migratory behavior in BC cells.

To further define the pathway by which FAM120A regulates progranulin-induced F-actin remodeling, we pharmacologically inhibited the small GTPases RhoA and Rac1, using specific small molecule inhibitors Rhosin and EHT-1864, respectively as in fact a putative link between FAM120A and RhoA has been previously reported [22]. In serum-free conditions, Rhosin-treated cells displayed organized F-actin networks. However, unlike control cells, they failed to undergo F-actin remodeling upon progranulin stimulation (Fig. 6C-D). In contrast, inhibition of Rac1 did not prevent progranulin-induced cytoskeletal changes (Fig. 6C-D). These results indicate that RhoA, but not Rac1, is required for progranulin-dependent F-actin cytoskeletal remodeling and further support a model in which FAM120A regulates BC cell motility by enabling RhoA-mediated F-actin network reorganization.

Lastly, consistent with these results, we showed that RhoA activation was significantly enhanced upon progranulin exposure of shCTR-T24 and 5637 cells, peaking at early time points of progranulin stimulation. However, RhoA activation was markedly reduced in FAM120A-depleted cells in both T24 and 5637 lines (Fig. 6E, F). Importantly, total RhoA protein levels were comparable between shCTR and shFAM120A samples, indicating that FAM120A does not regulate RhoA expression levels but is instead required for its activation in response to progranulin stimulation. Collectively, these findings establish FAM120A as a critical mediator of progranulin-induced RhoA activation, which in turn drives F-actin cytoskeletal remodeling. The impairment of RhoA activation in FAM120A-deficient cells provides a mechanistic explanation for the observed defects in F-actin filament reorganization, further supporting a functional link between FAM120A and RhoA-dependent cytoskeletal dynamics in BC cells.

### Progranulin induces F-actin network remodeling through ERK1/2 pathways

**Fig. 7:**
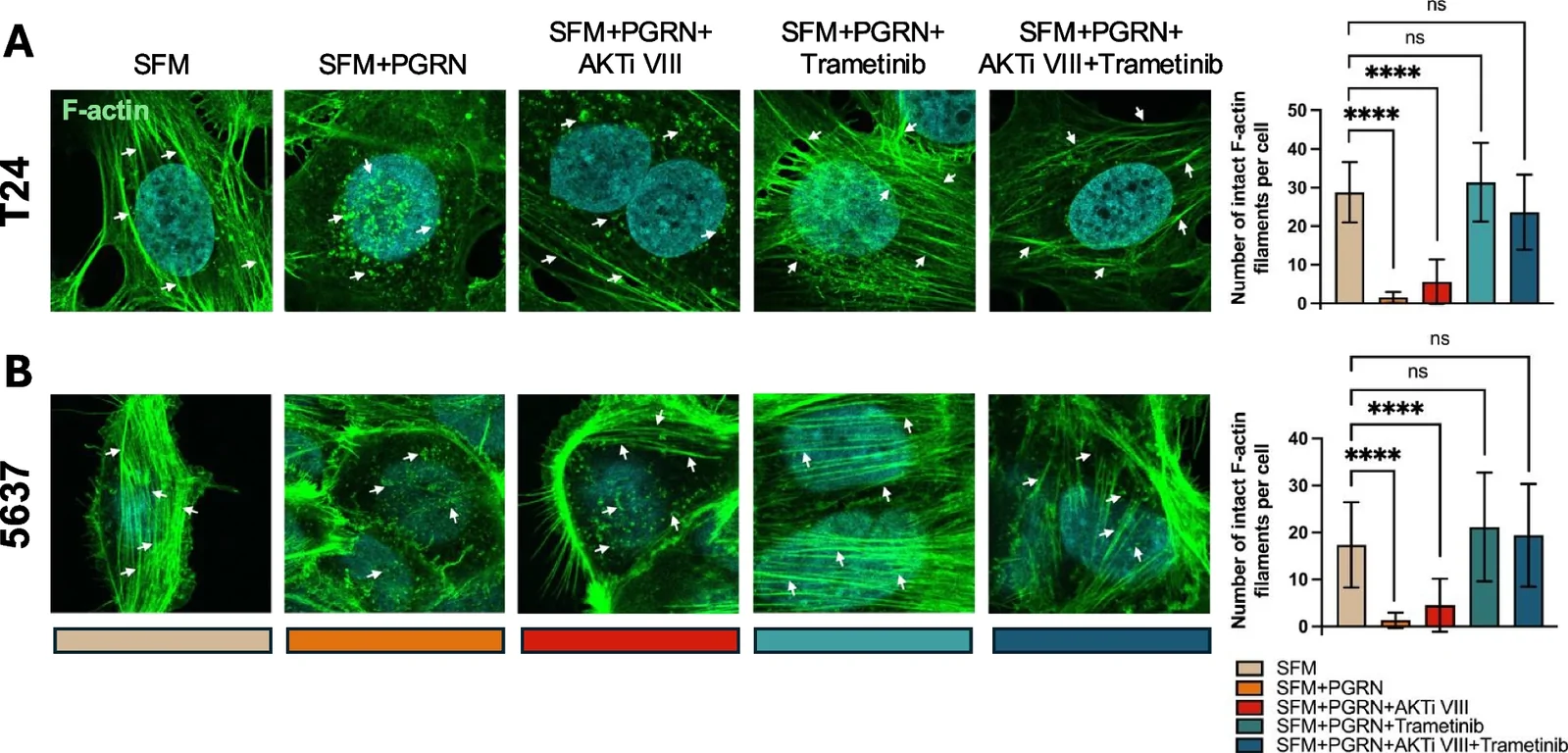
Progranulin induces F-actin network remodeling through ERK1/2 pathways. **A-B** T24 and 5637 cells were seeded on Ibidi plates, 20000 cells/chamber. The next day, cells were starved in SFM for 24 h. Cells were incubated with or without PGRN (progranulin) (100nM), PGRN + AKTi VIII (12.5 mM), or PGRN + Trametinib (100nM) or PGRN + AKTi VIII + Trametinib for 30 minutes, stained for F-actin using anti-phalloidin antibodies, and analyzed by immunofluorescence. Cell nuclei were stained with DAPI. Arrows point to intact F-actin or to F-actin filament remodeling. Quantification was performed using the ImageJ program. ns= not significant, *p < 0.05, **p < 0.01, ***p < 0.001 ****p < 0.0001

We previously showed that pharmacological inhibition of MEK significantly impaired progranulin- induced motility in BC [12]. Because FAM120A is required for progranulin-induced migration and invasion, we next investigated the signaling mechanisms underlying its regulation of F-actin cytoskeletal organization. As we demonstrated above, FAM120A depletion markedly attenuates progranulin-induced activation of both the AKT and ERK1/2 signaling pathways (Fig 3C, D). We therefore examined whether pharmacological inhibition of these two pathways affected F-actin remodeling evoked by progranulin stimulation. Serum-starved T24 and 5637 BC cells were stimulated without or with progranulin in the presence or absence of the AKT inhibitor, AKTi VIII, the ERK1/2 inhibitor, Trametinib, or the combination of both inhibitors, and F-actin remodeling was visualized by phalloidin staining and analyzed by confocal microscopy (Fig 7A, B). As shown before, stimulation with progranulin induced a significat reorganization of the F-actin cytoskeleton in both T24 and 5637 cells, characterized by the formation of abundant, well-organized stress fibers spanning the cell body (Fig. 7A, B). Inhibition of the AKT pathway partially disrupted stress fiber assembly, resulting in a sparse and disorganized F-actin network, whereas inhibition of ERK1/2 markedly disrupted the F-actin remodeling. The combined inhibition of both pathways produced a similar phenotype to ERK1/2 inhibition. Collectively, these findings demonstrated that progranulin-induced F-actin cytoskeletal remodeling depends prevalently on ERK1/2 activation while AKT might only partially contribute to this process. Together with our previous data showing that FAM120A is required for activation of these signaling cascades, these results support a model in which FAM120A functions upstream of AKT and ERK1/2 to promote actin stress fiber assembly, thereby facilitating the cytoskeletal changes necessary for BC cell migration and invasion.

### FAM120A is crucial for anchorage-independent and in vivo tumor growth in xenograft models

**Fig. 8:**
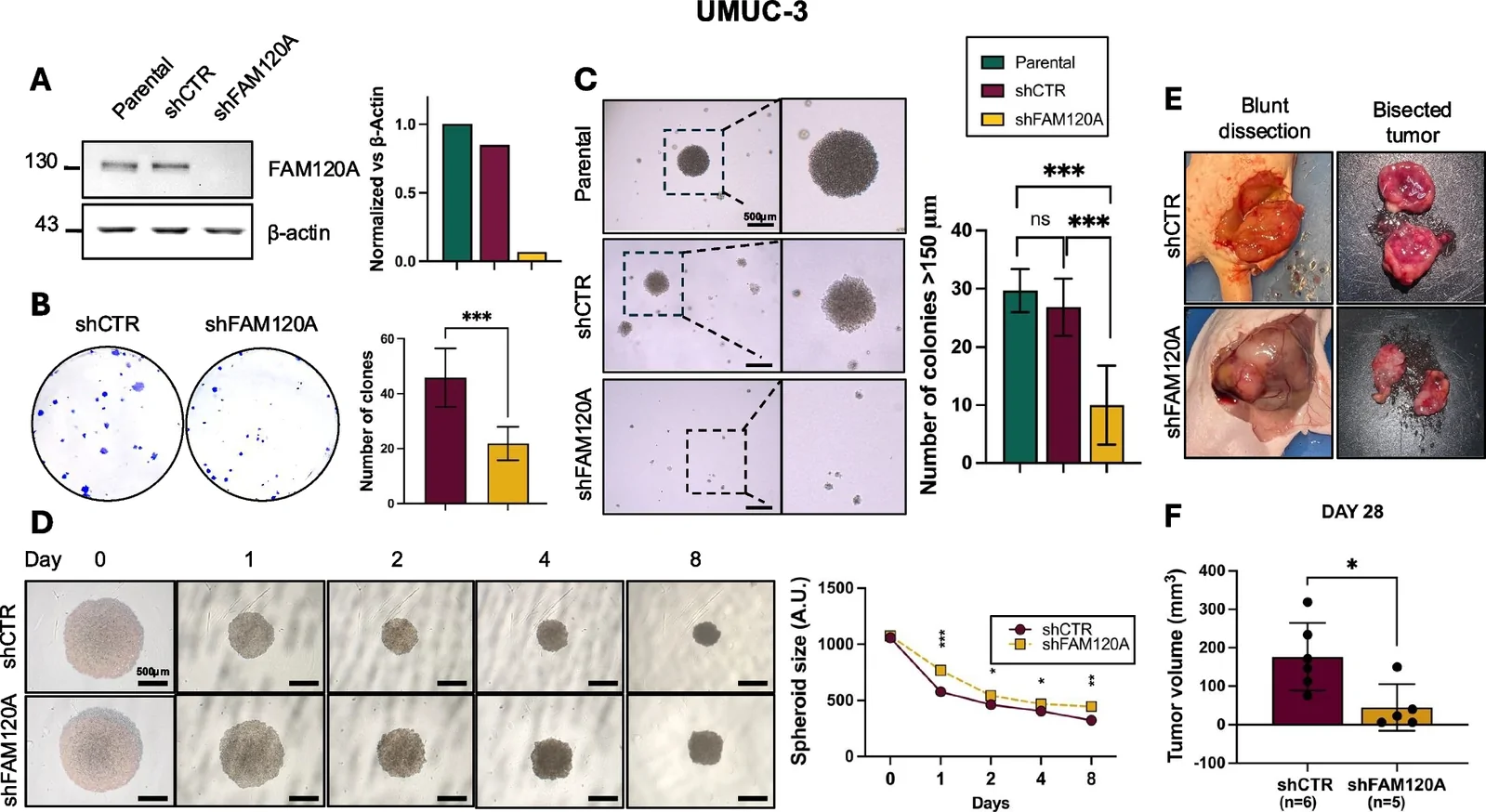
FAM120A is crucial for anchorage-independent growth and *in vivo* tumor growth in xenograft models. **A** Immunoblot confirming FAM120A-depletion in UMUC-3 cells. **B** Monolayer clonogenic assay performed in shCTR- and FAM120A-depleted UMUC-3 cells. Data shown are the average of 3 independent experiments performed in duplicate. **C** Anchorage-independent growth was assessed using the soft-agar colony formation assay. Briefly, 3000 cells were seeded onto a top layer containing 0.2% agar, whereas the bottom layer contained 0.4% agar. Colonies >150 mm were counted after 2 weeks. The assay was performed two times in triplicate. **D** Spheroid formation assay was performed in shCTR- and FAM120A-depleted UMUC-3 cells and monitored at the indicated time points. The spheroid’s size was measured using the ImageJ program. The data shown are the average of two experiments performed in at least 10 replicates. A.U.=Arbitrary Unit. **E-F** Tumor growth in nude mice subcutaneously implanted with shCTR or FAM120A-depleted UMUC-3 cells was measured at day 28. ns= not significant, *p < 0.05, **p < 0.01, ***p < 0.001 ****p < 0.0001

To further elucidate the role of FAM120A in BC progression, we evaluated the tumorigenic potential of FAM120A in modulating anchorage-independent growth and *in vivo* tumor formation. Notably, T24 and 5637 cells, although derived from invasive tumors, do not grow in anchorage independence and do not form tumors in mice [39, 40]. Thus, we depleted FAM120A in invasive-tumors-derived UMUC-3 cells, which express high levels of FAM120A (Fig. 2A) and have the ability to grow in anchorage independence in soft-agar assays and form tumors when injected into immunocompromised mice [14, 15, 19]. We achieved a significant depletion of FAM120A proteins in UMUC-3 cells *vis-à-vis* parental and control-transfected cells (Fig. 8A), which was associated with a significant inhibition of clonogenicity in monolayer of these cells, as compared to control cells (Fig. 8B) In addition, FAM120A-depleted UMUC-3 cells displayed a marked inhibition of colony formation in soft agar (Fig. 8C) and a reduction in their ability to form 3D spheroids (Fig. 8D) as compared to control-infected UMUC-3 cells (Fig. 8C, D). We previously demonstrated using both orthotopic and xenograft models that depletion of progranulin, EphA2, or drebrin, a F-actin-binding protein, in BC cells led to the inhibition of tumor formation in mice [14, 15, 19]. Thus, we compared the capacity of control- or FAM120A-depleted UMUC3 cells to promote *in vivo* tumor formation in xenograft models. Notably, FAM120A-depleted UMUC-3 cells were significantly inhibited in their *in vivo* tumorigenic ability compared to vehicle-infected UMUC-3 cells (Fig. 8E, F). UMUC-3-derived tumor tissues were highly hemorrhagic and necrotic, and we could not section them for further analysis. Collectively, these findings demonstrate that FAM120A is essential for BC tumorigenicity, as its depletion not only markedly impairs anchorage-independent growth but also significantly reduces *in vivo* tumor formation.

### FAM120A depletion sensitizes BC cells to cisplatin treatment

**Fig. 9:**
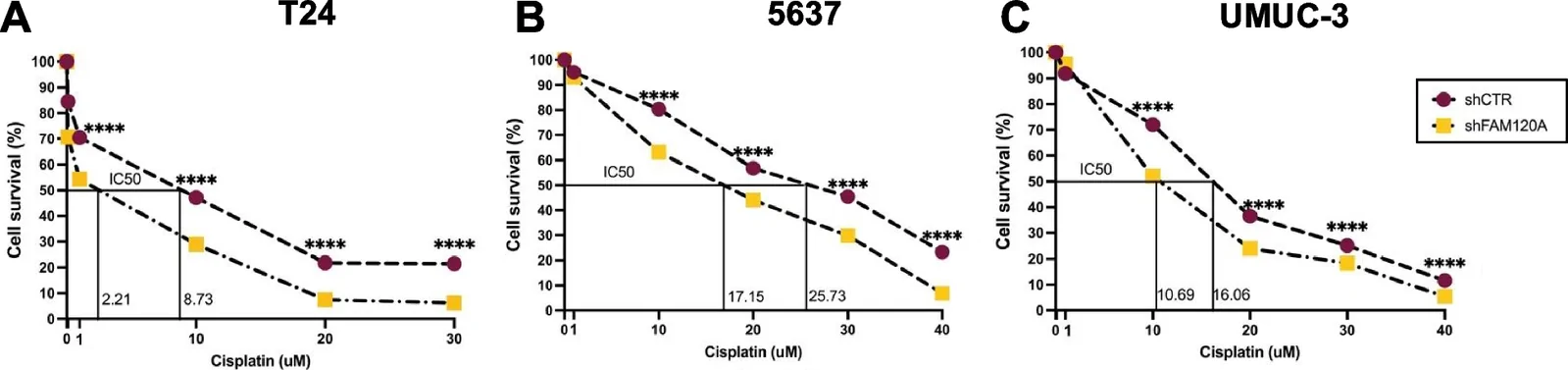
FAM120A depletion sensitizes BC cells to cisplatin treatment. **A-C** Sensitivity of shCTR- and shFAM120A-depleted T24, 5637, or UMUC-3 cells to the drug was measured using various concentrations of cisplatin. Mean ±SD; *n* = 2 biological replicates run in 5 replicates. ns= not significant, *p < 0.05, **p < 0.01, ***p < 0.001 ****p < 0.0001.

We previously demonstrated that progranulin [15] and EphA2 [19] depletion sensitized UMUC-3 cells to cisplatin treatment. To determine whether FAM120A influences chemosensitivity to cisplatin, we evaluated cell viability upon exposure to increasing concentrations of cisplatin in T24, 5637, and UMUC-3 BC cell lines. In all cell lines, cisplatin treatment resulted in dose-dependent cell death of both shCTR and FAM120A-depleted cells. However, FAM120A depletion significantly enhanced sensitivity to cisplatin, as evidenced by a significant reduction in cell viability at nearly all tested concentrations compared to controls (Fig. 9A, B, C). Consistent with this observation, the half-maximal inhibitory concentration (IC50) values were markedly decreased in FAM120A-depleted cells relative to shCTR cells across all cell lines. Collectively, these results suggest that FAM120A may contribute to chemoresistance and highlight its potential as a therapeutic target to enhance cisplatin efficacy.

## Discussion

BC is a major global health issue and has been ranked the ninth most common type of cancer worldwide and the seventh in the United States [41]. Non‐muscle‐invasive BCs present high rates of recurrence and progression, as in fact a study has shown that nearly three‐quarters of patients with high‐risk disease will recur, progress, or die within 10 years [42]. Moreover, progranulin is upregulated in BC tissues, and *GRN* upregulation correlated with decreased overall survival [12]. Progranulin is a secreted glycoprotein that serves as a pluripotent growth factor and promotes tumor cell angiogenesis, migration, tumorigenesis, and proliferation [3, 17, 43–45], and its dysregulation in cancer affects both tumor initiation and progression [46]. In addition to its direct effects on tumor cells, progranulin has emerged as a critical regulator of the tumor microenvironment, where it modulates inflammatory responses, influences immune cell recruitment and activation, and contributes to the establishment of a pro-tumorigenic niche that supports cancer progression [47].

**Fig. 10:**
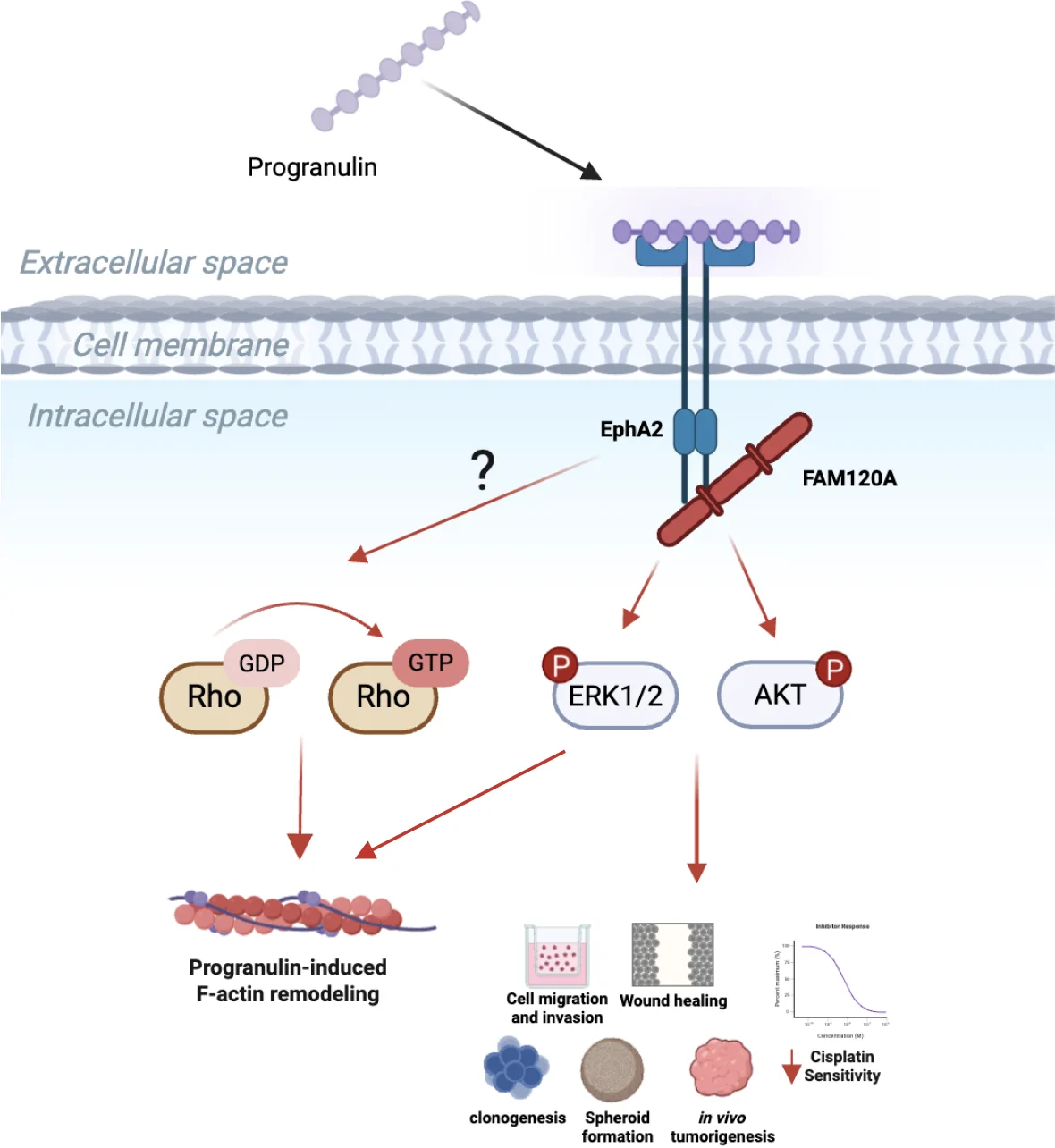
Schematic representation of progranulin/EphA2/FAM120A axis signaling in BC

We recently performed MS/LC analysis on BC cells [19] and identified several progranulin-dependent EphA2 interactor proteins, including FAM120A, which plays a role in promoting cancer cell proliferation and growth, enhancing resistance to chemotherapy, and metastasis [21, 22, 29]. In this study, we demonstrate that: 1) FAM120A is upregulated in BC tissues as compared to normal control, and its expression correlated with EphA2. In addition, High FAM120A expression levels are associated with poor survival. 2) FAM120A is well-expressed in various BC cell lines derived from low to high-grade urothelial carcinomas and is a progranulin-dependent EphA2 interactor required for progranulin-induced activation of AKT and ERK1/2 pathways; 3) FAM120A modulates progranulin-evoked BC cell migration, invasion, clonogenic capacity, anchorage-independent growth, 3D spheroid formation, and *in vivo* tumor formation; 4) FAM120A depletion inhibits progranulin-induced F-actin cytoskeleton remodeling in BC cells through a RhoA-dependent pathway. 5) Progranulin induces F-actin remodeling mainly through ERK1/2 and partially through AKT pathways. Our data expand the current understanding of progranulin/EphA2 oncogenic signaling by identifying and functionally characterizing FAM120A as a novel progranulin-dependent EphA2 interactor that contributes to BC formation (Fig. 10).

Proteomic analysis of the EphA2 interactome revealed a wide network of progranulin-mediated interactions, among which FAM120A emerged as a previously unidentified binding partner. These data add a new layer of complexity to the progranulin/EphA2 signaling pathway, which has been implicated in regulating tumor cell motility, invasion, and survival across multiple malignancies [19]. FAM120A, a scaffold protein with poorly characterized function, is encoded by the *FAM120A* gene located on chromosome 9q22.31. It is mainly an intracellular protein and is found in the cytosol [48]. FAM120A links lipogenic gene transcription and splicing to tumor growth [21]. It was previously demonstrated that FAM120A can bind to SLC7A11 mRNA, enhancing its stability, thereby inhibiting ferroptosis in gastric cancer cells [29]. In addition, FAM120A is involved in the migration and invasion of colon cancer cells through IL12Rα2 [22]. FAM120 expression is significantly upregulated in BC tissues as compared to benign or adjacent tissue controls and a survival analysis, generated using the DriverDBv4 platform [30], demonstrated that patients with high *FAM120A* expression exhibited a significantly shorter survival duration compared with those expressing low levels of *FAM120A* (log-rank *P =* 0.0221), with a hazard ratio (HR) of 1.42. This indicates that patients with elevated *FAM120A* expression have an approximately 42% greater risk of death than those with lower expression levels, suggesting that FAM120A may serve as a clinically relevant diagnostic and possibly prognostic biomarker in BC. Progranulin enhanced the EphA2-FAM120A interaction, confirmed by co-immunoprecipitation, colocalization, and PLA, supporting a ligand-dependent role for FAM120A in EphA2 signaling, thereby providing robust evidence that FAM120A is potentially critical for progranulin/EphA2 downstream oncogenic signaling.

As the primary ligand of EphA2, EphrinA1 binding triggers receptor autophosphorylation on tyrosine residues (*canonical* signaling), thereby promoting its activation [49–51] followed by endocytic internalization and subsequent lysosomal degradation, thereby inhibiting EphA2 oncogenic signaling. [27, 52, 53].

We previously showed that progranulin binding to EphA2 triggers non-canonical signaling via Tyr588 phosphorylation, activation of AKT and ERK1/2 and a positive feedback loop that promotes Ser897 phosphorylation and BC cell motility [19]. Here, we found that loss of FAM120A compromised progranulin-evoked activation of AKT and ERK1/2 pathways, two central signaling cascades known to promote tumor cell survival, proliferation, and resistance to therapy [54–56]. The requirement of FAM120A for full activation of AKT and ERK1/2 positions it as a critical signaling hub linking EphA2 receptor engagement to the activation of major oncogenic pathways. Rather than representing independent mechanisms, our findings support a model in which FAM120A links progranulin/EphA2 signaling to AKT/ERK1/2 activation, leading to RhoA-dependent F-actin cytoskeletal remodeling. By promoting RhoA-dependent F-actin reorganization, FAM120A enables BC cells to acquire the dynamic cytoskeletal architecture necessary for efficient migration and invasion. These findings suggest that impaired cytoskeletal plasticity provides the mechanistic basis for the reduced migratory and invasive phenotype observed following FAM120A depletion. Because dynamic actin reorganization is a critical step in directional cell migration and invasion, we suggest that cytoskeletal remodeling is the central mechanism underlying FAM120A function in BC.

In addition to its role in intracellular signaling, FAM120A contributes to the migratory and invasive capacities of BC cells. Progranulin and EphA2 are key regulators of migration, invasion, and proliferation in various tumor cell models [17, 44, 45, 57–61], and we previously showed that the progranulin/EphA2 axis promotes BC cell migration and invasion [19]. In the present study, we demonstrated that depletion of FAM120A markedly impaired progranulin-driven motility and invasion, underscoring its requirement for these progranulin-evoked phenotypes. However, it is worth mentioning that FAM120A depletion also mildly affected progranulin-independent motility, suggesting that FAM120A may have a broader role in motility and contribute to additional oncogenic pathways in BC.

The concomitant disruption of progranulin-mediated actin cytoskeletal remodeling further supports the idea that FAM120A regulates cytoskeletal dynamics and maybe focal adhesion turnover, as in fact published data suggest that FAM120A may mediate IL13Rα2-induced activation of focal adhesion kinase (FAK) [22]. However, we don’t know at the moment whether FAM120A plays a role in modulating progranulin-dependent focal adhesion assembly/disassembly, as we previously demonstrated in mesothelioma-derived cell lines [17, 36]. These findings support our observation that FAM120A depletion impairs F-actin remodeling downstream of the progranulin/EphA2 axis, suggesting that FAM120A coordinates signaling from the cell membrane to promote cytoskeletal reorganization and directional migration. To further elucidate the signaling mechanisms through which FAM120A governs progranulin-induced cytoskeletal remodeling, we examined the involvement of key GTPases critical for cell motility [62, 63] using pharmacological inhibitors of RhoA and Rac1. Interestingly, RhoA inhibition preserved organized F-actin structures under serum-free conditions but completely abolished cytoskeletal rearrangements triggered by progranulin, indicating that RhoA activity is essential for progranulin/EphA2/FAM120A-dependent F-actin remodeling. In contrast, inhibition of Rac1 did not impair this process, suggesting that this GTPase is dispensable for progranulin-dependent regulation of F-actin remodeling. These observations identify RhoA as the predominant regulator of progranulin-driven F-actin dynamics and support a model in which FAM120A promotes BC cell motility by enabling RhoA-mediated cytoskeletal reorganization. The observation that FAM120A depletion markedly attenuated progranulin-induced RhoA activation in both T24 and 5637 cells suggests that FAM120A functions upstream of RhoA within the progranulin signaling network. Importantly, total RhoA protein levels were unaffected by FAM120A depletion, indicating that FAM120A regulates RhoA activation rather than its expression. Collectively, these findings highlight FAM120A as a central coordinator of progranulin-responsive F-actin network remodeling, integrating upstream signaling cues to facilitate the directional migration and invasive behavior characteristic of aggressive BC cells.

Our findings also demonstrate a broader role of FAM120A in promoting tumorigenic phenotypes, as in fact, FAM120A depletion significantly reduced anchorage-independent growth, impaired 3D spheroid formation, and inhibited *in vivo* tumor formation. These results identify FAM120A as a key regulator of bladder tumor initiation, reinforcing the notion that progranulin/EphA2 signaling drives multiple hallmarks of malignancy. The loss of tumorigenic potential following FAM120A depletion underscores its role in coordinating oncogenic signaling and cytoskeletal remodeling to promote tumorigenesis. Whether FAM120A also contributes to BC progression and metastasis remains to be determined.

Recent studies show that FAM120A acts downstream of mTORC1 as a transcriptional coactivator and splicing regulator, coordinating lipogenic gene expression through SRSF1 [21]. By promoting lipid synthesis, FAM120A supports the metabolic demands of cancer cell proliferation and may also facilitate tumor growth under stress conditions, including 3D spheroid formation and anchorage-independent growth. Moreover, FAM120A has been implicated in cisplatin resistance and cellular stress responses in gastric and non-small cell lung cancers [20, 29]. In the present study, FAM120A depletion enhanced cisplatin treatment response and significantly decreased IC₅₀ values compared to control cells. The ability of FAM120A depletion to enhance cisplatin sensitivity highlights its potential as a therapeutic target to improve cisplatin efficacy in advanced BC. Although no therapies currently target FAM120A, several EphA2-directed agents, including monoclonal antibodies [27, 64], antibody-drug conjugates [65], bicycle drug conjugates [66], PROTACs [67], nanoparticles [68] and CAR-T cells [69], have entered early-phase or phase I clinical trials. There are anti-progranulin antibody (AG01) [3, 70] and anti-sortilin1antibody [71] to reduce progranulin levels in a first-in-human phase I clinical trial. Our findings suggest that disrupting the downstream scaffold protein FAM120A may represent an alternative strategy to inhibit EphA2-driven oncogenic signaling while enhancing cisplatin sensitivity in BC.

Collectively, our findings establish FAM120A as a key effector of progranulin/EphA2 signaling in BC, linking EphA2 activation to AKT/ERK1/2 signaling and cytoskeletal remodeling to promote transformation. This is consistent with studies in mesothelioma demonstrating that progranulin activates multiple receptor pathways to drive oncogenic signaling [17, 36]. Together, these findings highlight the versatility of progranulin as a multifunctional growth factor that engages distinct receptor networks to promote tumorigenesis. Given its association with aggressive disease, FAM120A represents a promising biomarker and therapeutic target in BC. Future studies should define the structural basis and signaling mechanisms of the EphA2-FAM120A complex to identify new therapeutic opportunities.

## Conclusion

This study identifies FAM120A as a novel, progranulin-dependent EphA2 interactor that plays a pivotal role in BC progression. We demonstrate that FAM120A is overexpressed in BC tissues and required for progranulin-induced activation of AKT and ERK1/2 signaling, cytoskeletal remodeling, and the promotion of cell migration and invasion. Moreover, loss of FAM120A markedly reduces clonogenic growth, 3D spheroid formation, and *in vivo* tumorigenicity, underscoring its essential role in sustaining malignant phenotypes. FAM120A depletion sensitizes the BC cells to cisplatin treatment. Collectively, these findings establish FAM120A as a key effector of the progranulin/EphA2 axis and suggest that targeting FAM120A may represent a promising therapeutic approach for BC treatment.

## Supplementary Information

Additional file 1: Supplementary Fig. 1. FAM120A-depletion in 5637 cells and clonogenic, migration, and invasion assay. A) Immunoblot showing FAM120A-depletion in 5637 cells using a second different shFAM120A plasmid. B) Migration and C) Invasion through Matrigel were assessed as described in the Fig. 4 legend. D) Monolayer clonogenic assay performed in shCTR- and FAM120A-depleted 5637 cells. The data presented are the average of two independent experiments run in triplicate. ***p < 0.001.

Additional file 2: Uncropped blots of images are provided

Additional file 3: MS/LC analysis containing the EphA2 interactors

Additional file 4: Raw data files containing EphA2 or FAM120A staining quantification and TMA sample information

## Data Availability

The data that support the findings of this study are available from the corresponding author upon reasonable request.

## Abbreviations

EGFR: epidermal growth factor receptor
EphA2: erythropoietin-producing hepatocellular carcinoma receptor A2
ERK: extracellular signal-regulated kinase
FAM120A: Family with Sequence Similarity 120 Member A
IL13Rα2: Interleukin-13 receptor subunit alpha-2
RYK: receptor-like tyrosine kinase
SRSF1: Serine/Arginine-Rich Splicing Factor 1
