# Supplementary material for: Functional characterization of FAM120A as a novel effector in the progranulin/EphA2 oncogenic axis in bladder cancer
Source: J Exp Clin Cancer Res. 2026 Aug 11;45:205. doi: 10.1186/s13046-026-03808-1 (PMC13602547; doi:10.1186/s13046-026-03808-1)
Supplement: Supplementary file 1 [file 13046_2026_3808_MOESM2_ESM.pdf]

WB: FAM120A

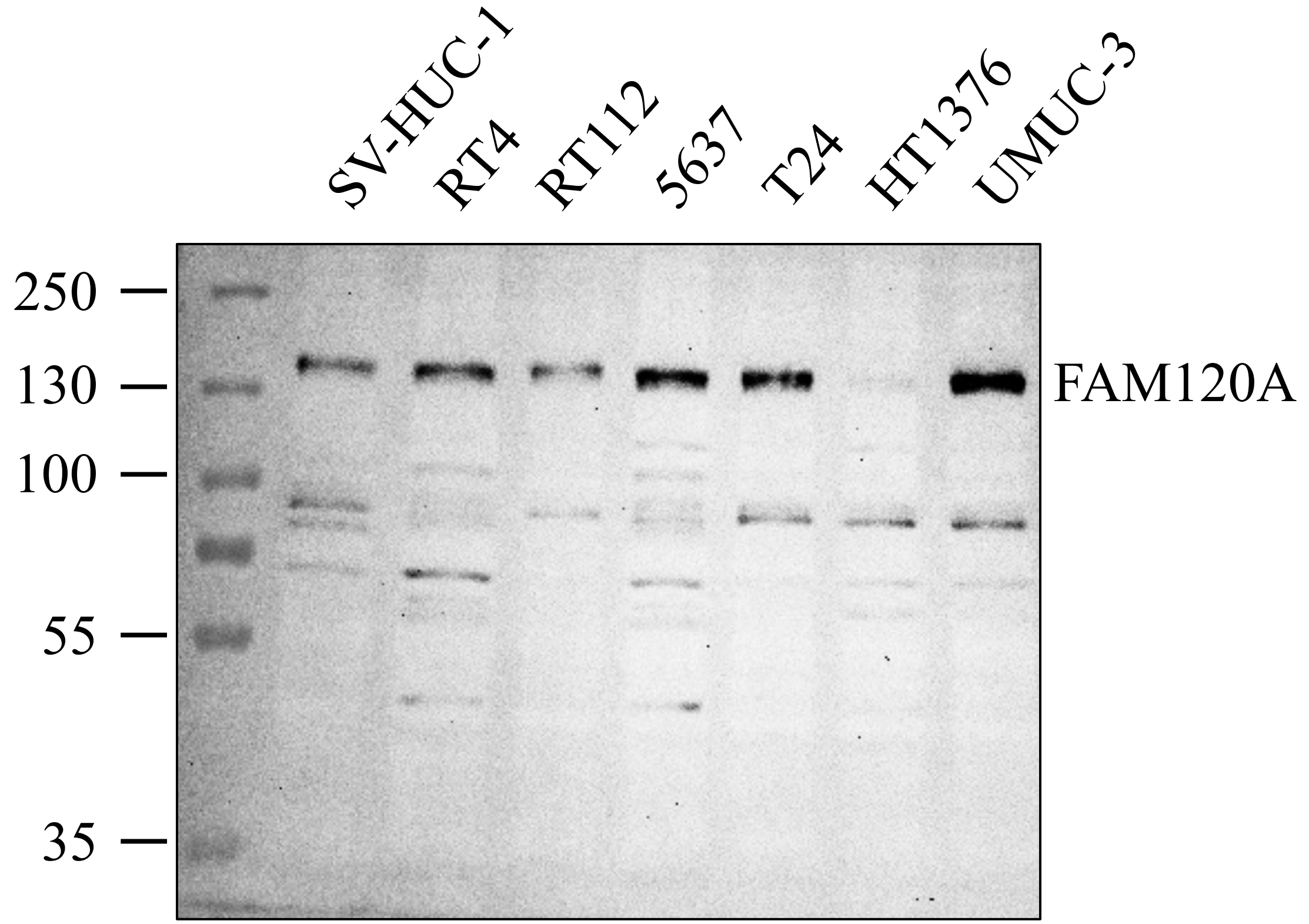

SV-HUC-1
RT4
RT112
5637
T24
HT1376
UMUC-3
250
130
100
55
35
FAM120A

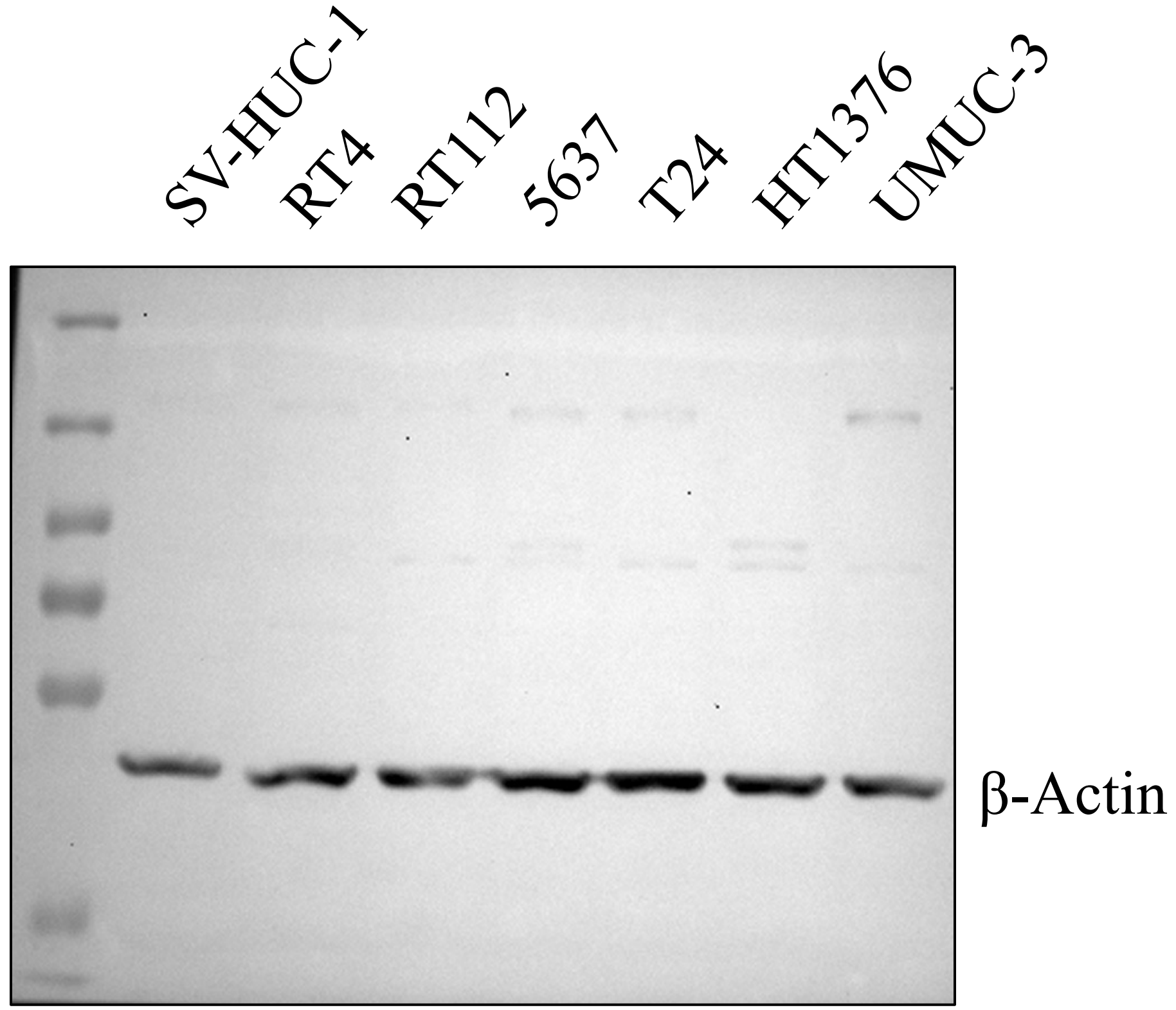

SV-HUC-1
RT4
RT112
5637
T24
HT1376
UMUC-3
β-Actin

IP: EphA2 WB: FAM120A

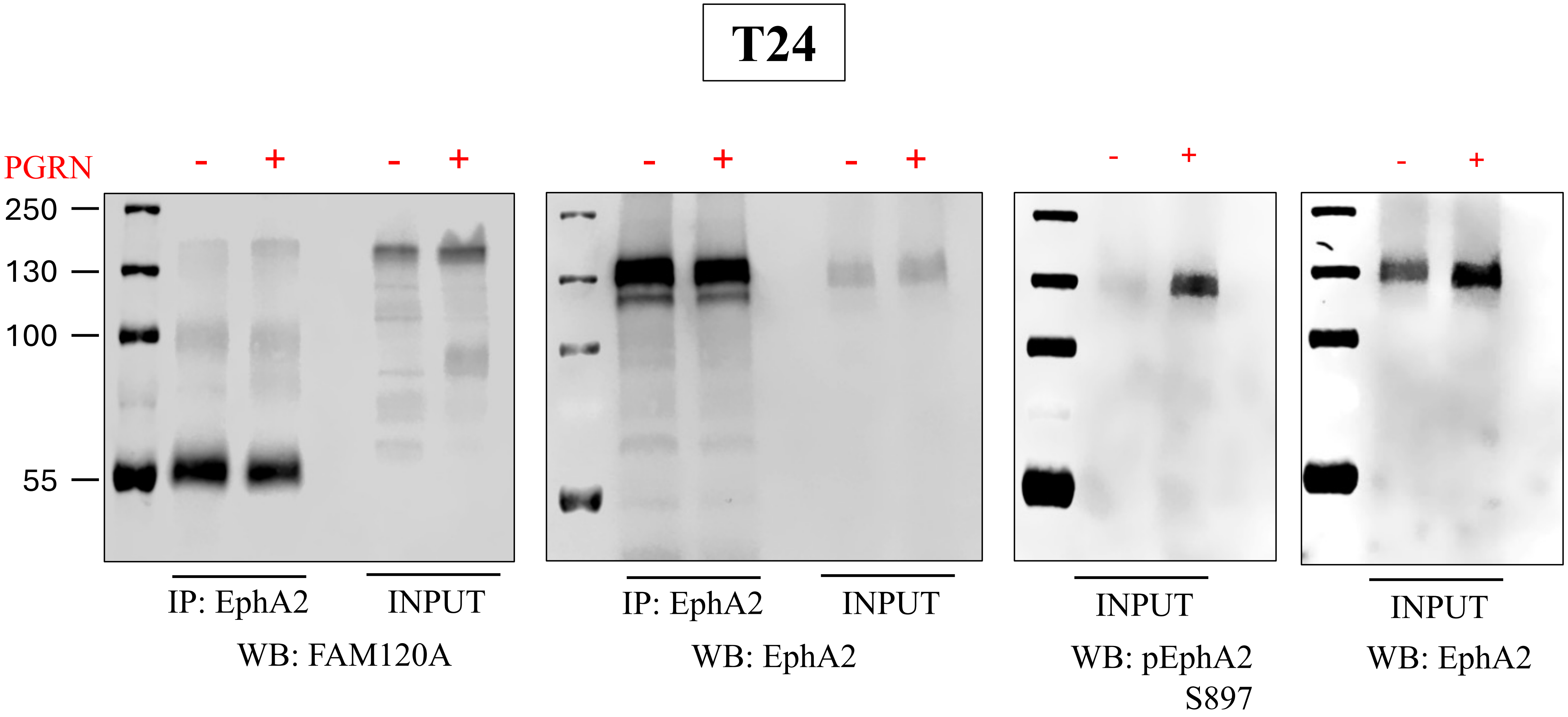

IP: FAM120A WB: EphA2

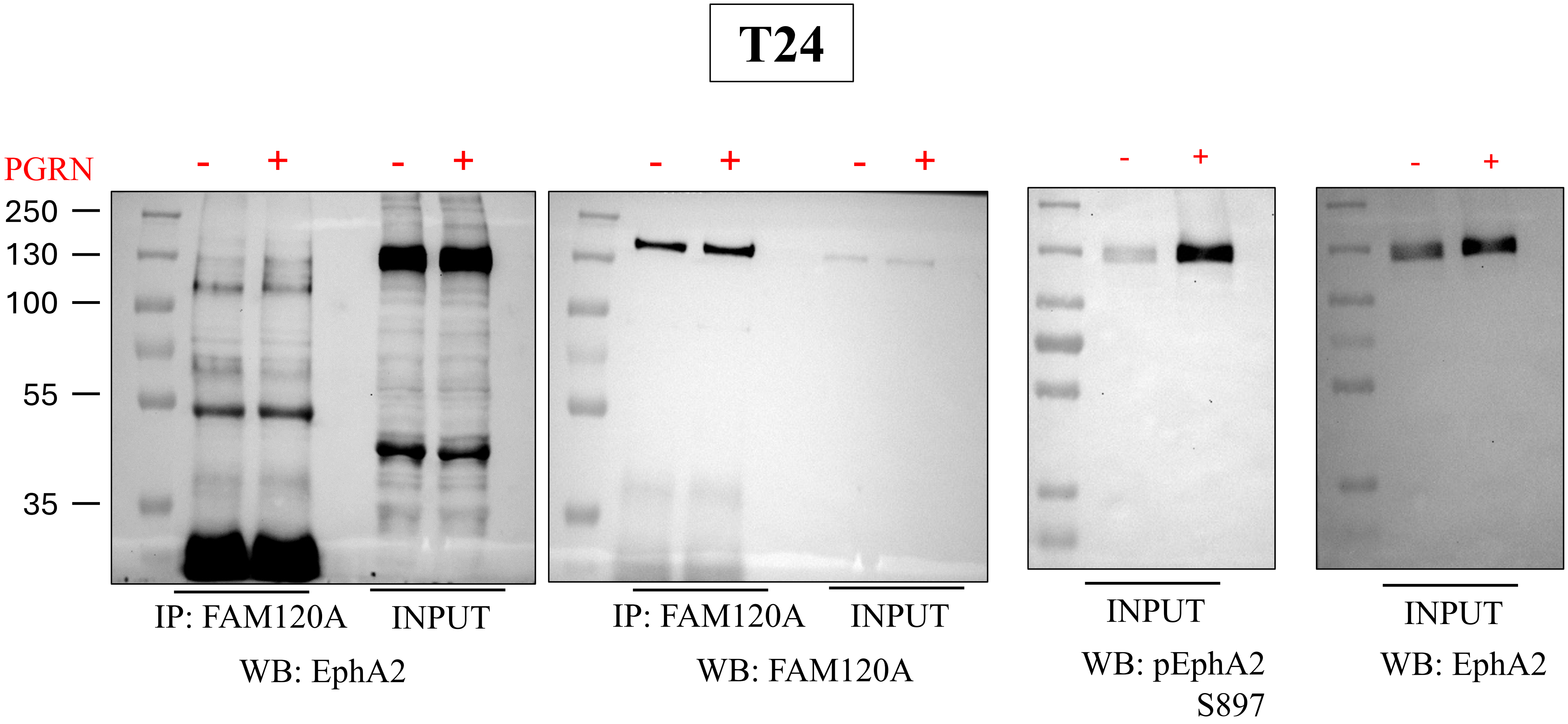

WB: FAM120A

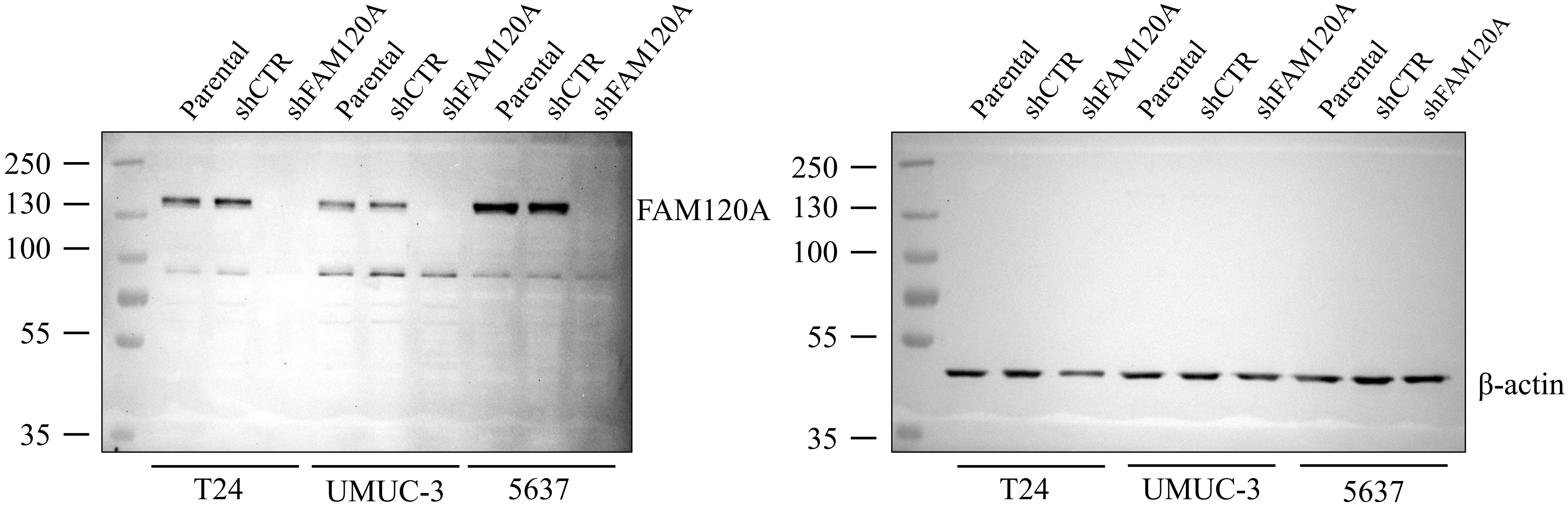

Parental
shCTR
shFAM120A
Parental
shCTR
shFAM120A
Parental
shCTR
shFAM120A
250
130
100
55
35
FAM120A
T24
UMUC-3
5637
Parental
shCTR
shFAM120A
Parental
shCTR
shFAM120A
Parental
shCTR
shFAM120A
250
130
100
55
35
β-actin
T24
UMUC-3
5637

WB: FAM120A

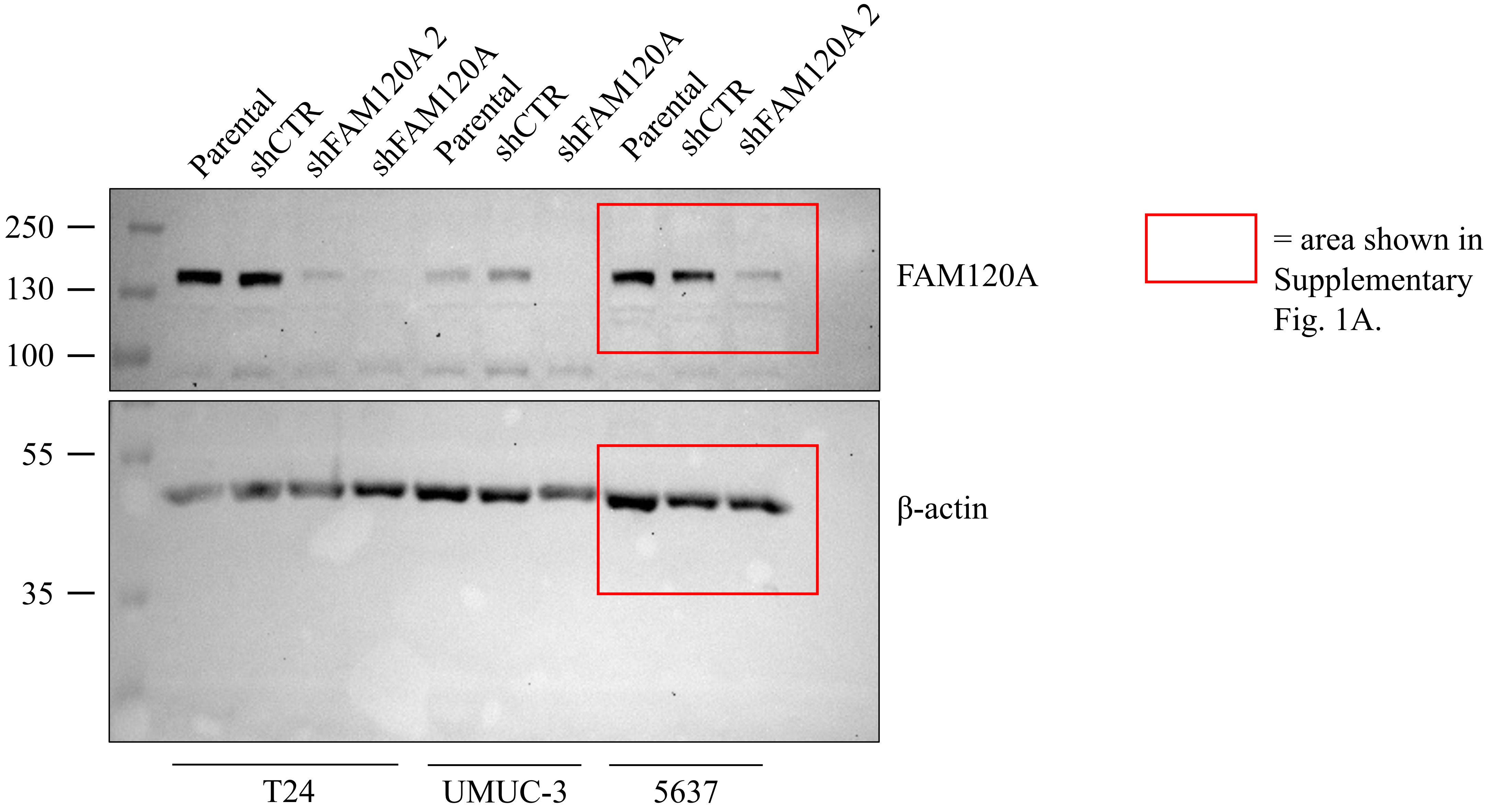

Parental
shCTR
shFAM120A 2
shFAM120A
Parental
shCTR
shFAM120A
Parental
shCTR
shFAM120A 2
250
130
100
55
35
FAM120A
β-actin
T24
UMUC-3
5637
= area shown in Supplementary Fig. 1A.

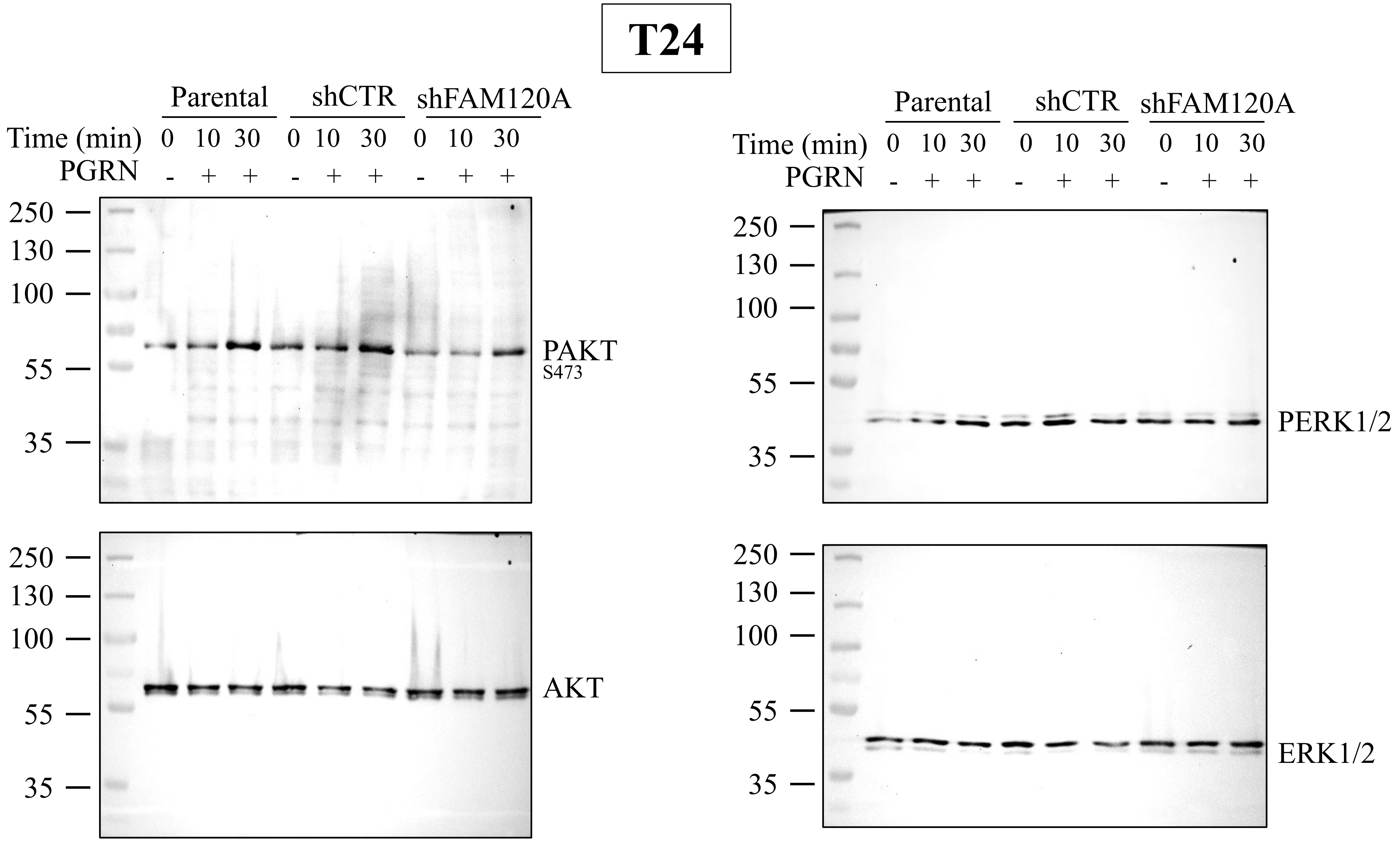
T24
Parental
shCTR
shFAM120A
Time (min)
0 10 30 0 10 30 0 10 30
PGRN
- + + - + + - + +
250
130
100
55
35
PAKT
S473
AKT
PERK1/2
ERK1/2

5637

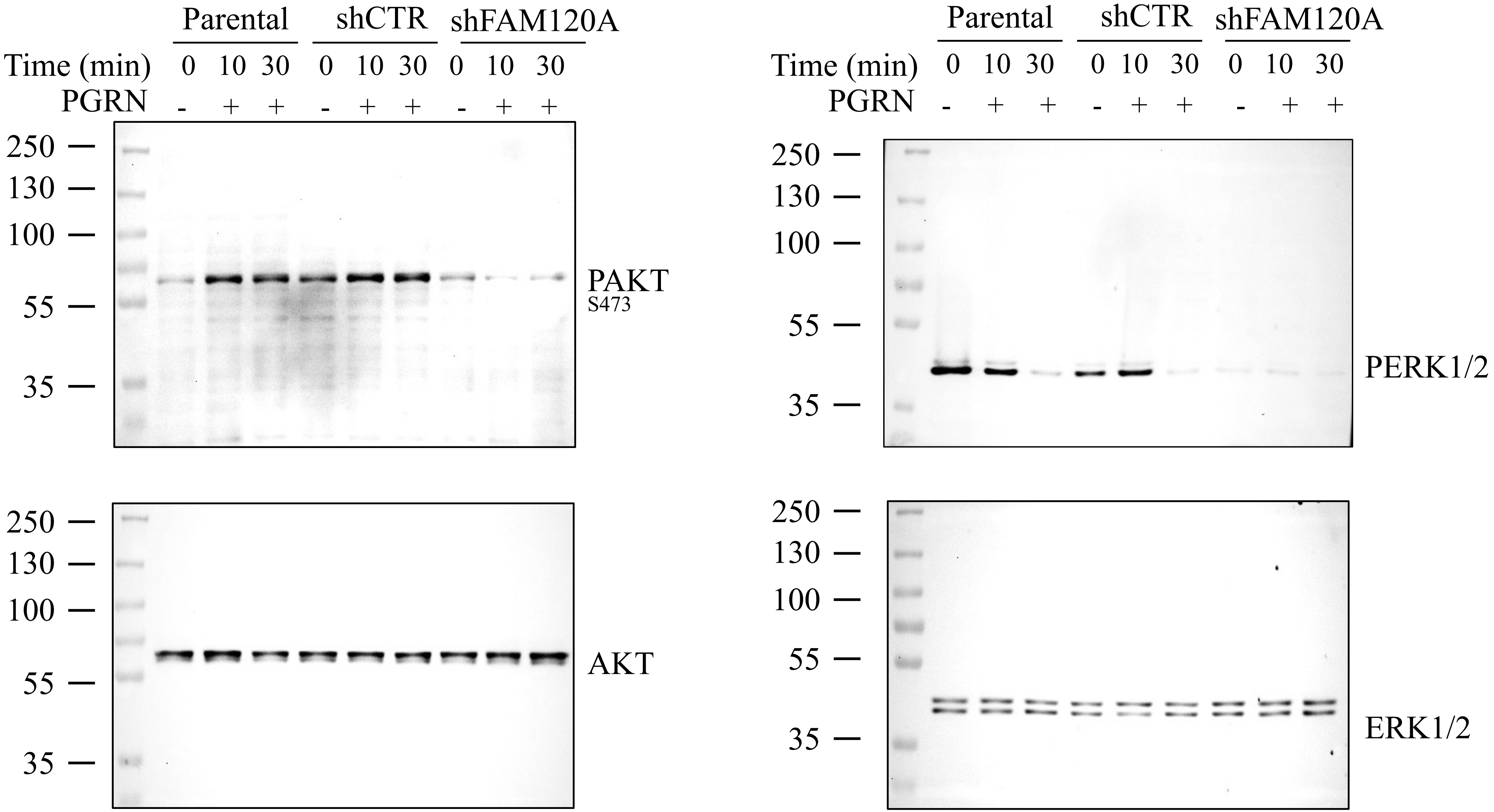
Parental
shCTR
shFAM120A
Time (min) 0 10 30 0 10 30 0 10 30
PGRN - + + - + + - + +
250
130
100
55
35
PAKT
S473
AKT
PERK1/2
ERK1/2

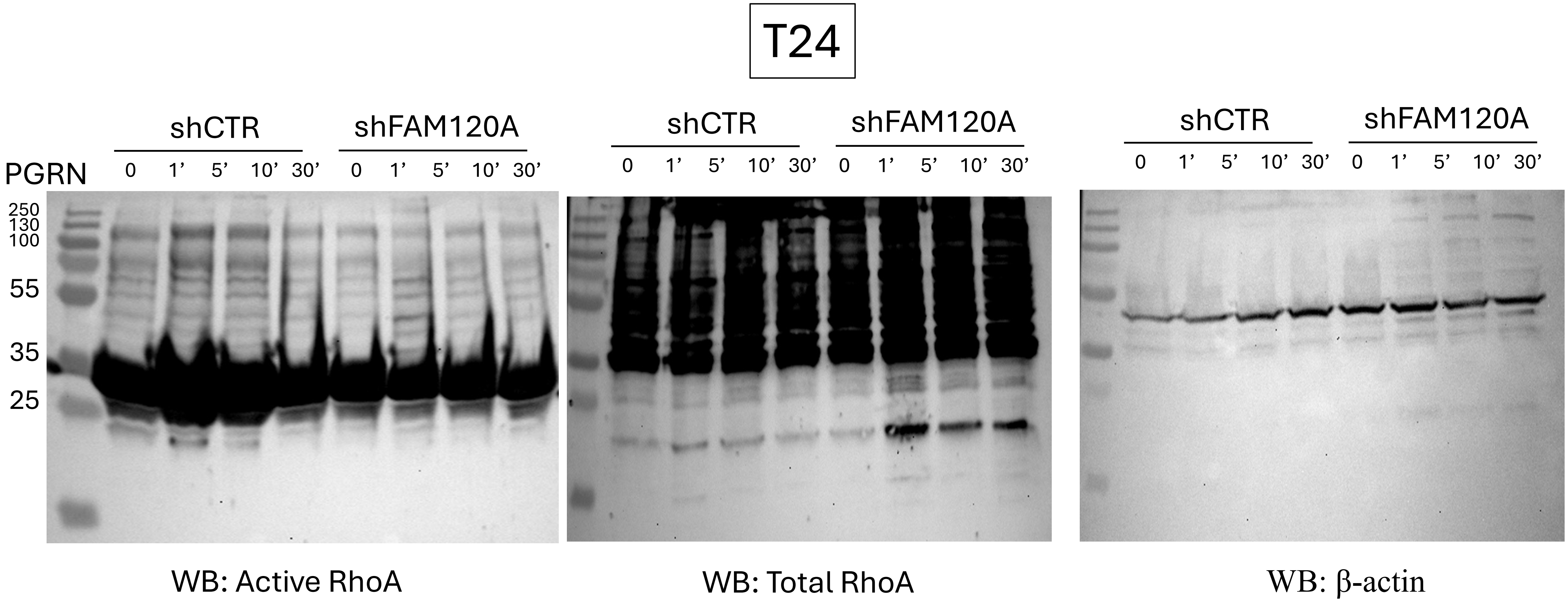
T24
shCTR
shFAM120A
PGRN
0 1’ 5’ 10’ 30’ 0 1’ 5’ 10’ 30’
250
130
100
55
35
25
WB: Active RhoA
shCTR
shFAM120A
0 1’ 5’ 10’ 30’ 0 1’ 5’ 10’ 30’
WB: Total RhoA
shCTR
shFAM120A
0 1’ 5’ 10’ 30’ 0 1’ 5’ 10’ 30’
WB: β-actin

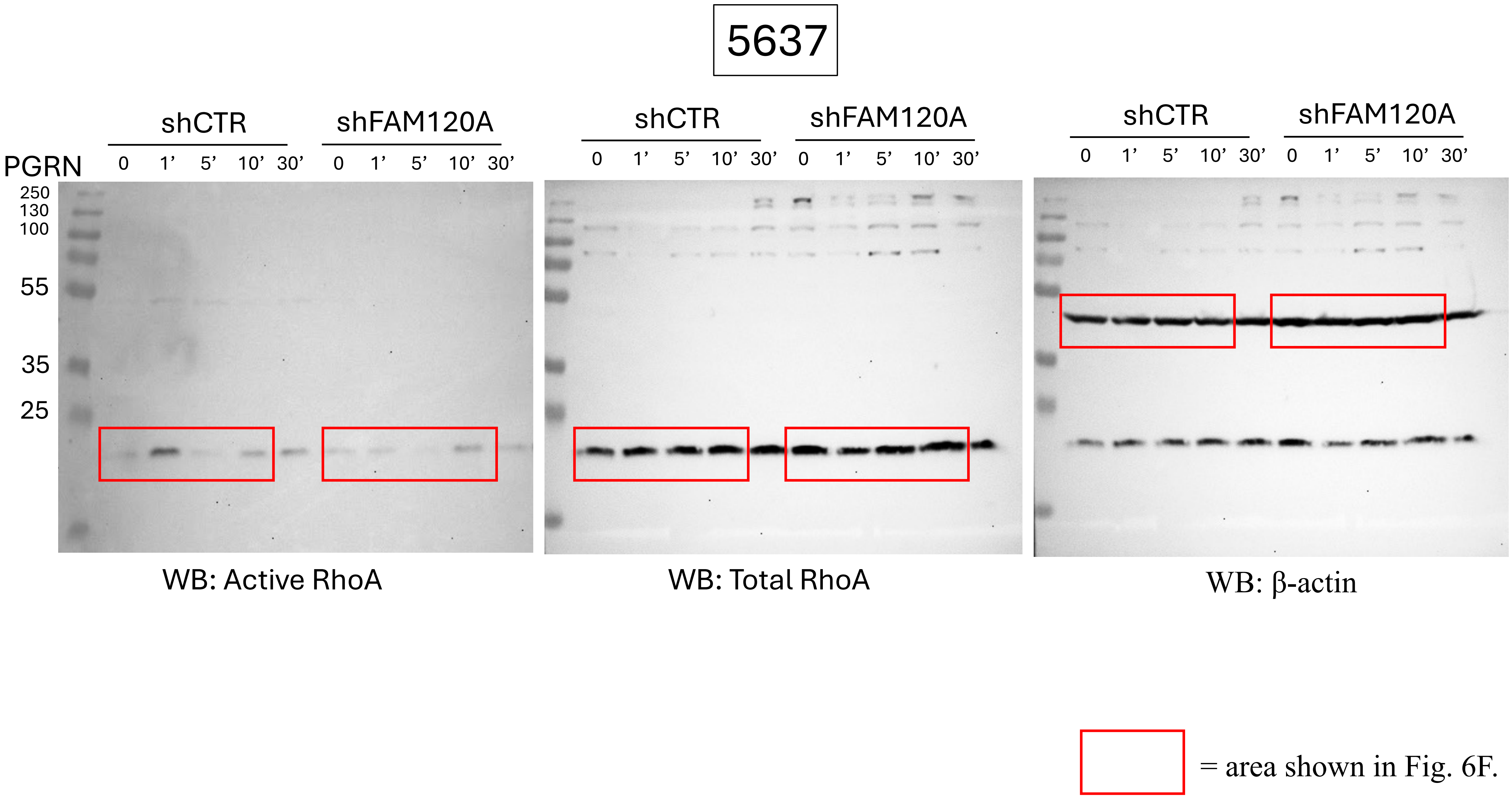
5637
shCTR
shFAM120A
PGRN
0
1'
5'
10'
30'
250
130
100
55
35
25
WB: Active RhoA
WB: Total RhoA
WB: β-actin
= area shown in Fig. 6F.
