# Supplementary material for: Functional characterization of FAM120A as a novel effector in the progranulin/EphA2 oncogenic axis in bladder cancer
Source: J Exp Clin Cancer Res. 2026 Aug 11;45:205. doi: 10.1186/s13046-026-03808-1 (PMC13602547; doi:10.1186/s13046-026-03808-1)
Supplement: Supplementary file 2 [file 13046_2026_3808_MOESM1_ESM.docx]

**5637**

130

43

FAM120A

β

-

actin

**C**

**D**

**A**

**B**

shCTR

SFM

SFM+PGRN

**Invasion**

shCTR

shFAM120A #2

SFM

SFM+PGRN

**Migration**

shCTR

shFAM120A #2

shFAM120A #2

**Supplementary Figure 1**
